# α-Synuclein conformational strains spread, seed and target neuronal cells differentially after injection into the olfactory bulb

**DOI:** 10.1186/s40478-019-0859-3

**Published:** 2019-12-30

**Authors:** Nolwen L. Rey, Luc Bousset, Sonia George, Zachary Madaj, Lindsay Meyerdirk, Emily Schulz, Jennifer A. Steiner, Ronald Melki, Patrik Brundin

**Affiliations:** 1 Center for Neurodegenerative Science, Van Andel Institute, 333 Bostwick Avenue N.E, Grand Rapids, MI 49503 USA; 2 Institut François Jacob (MIRCen), CEA and Laboratory of Neurodegenerative diseases, UMR 9199 CNRS, 18 route du Panorama, 92265 Fontenay-aux-Roses, France; 3 Bioinformatics and Biostatistics Core, Van Andel Institute, 333 Bostwick Avenue N.E, Grand Rapids, MI 49503 USA

**Keywords:** Alpha-synuclein, Strains, Fibrils, Prion-like spreading, Olfactory bulb

## Abstract

Alpha-synuclein inclusions, the hallmarks of synucleinopathies, are suggested to spread along neuronal connections in a stereotypical pattern in the brains of patients. Ample evidence now supports that pathological forms of alpha-synuclein propagate in cell culture models and in vivo in a prion-like manner. However, it is still not known why the same pathological protein targets different cell populations, propagates with different kinetics and leads to a variety of diseases (synucleinopathies) with distinct clinical features. The aggregation of the protein alpha-synuclein yields different conformational polymorphs called strains. These strains exhibit distinct biochemical, physical and structural features they are able to imprint to newly recruited alpha-synuclein. This had led to the view that the clinical heterogeneity observed in synucleinopathies might be due to distinct pathological alpha-synuclein strains.

To investigate the pathological effects of alpha-synuclein strains in vivo, we injected five different pure strains we generated de novo (fibrils, ribbons, fibrils-65, fibrils-91, fibrils-110) into the olfactory bulb of wild-type female mice. We demonstrate that they seed and propagate pathology throughout the olfactory network within the brain to different extents. We show strain-dependent inclusions formation in neurites or cell bodies. We detect thioflavin S-positive inclusions indicating the presence of mature amyloid aggregates.

In conclusion, alpha-synuclein strains seed the aggregation of their cellular counterparts to different extents and spread differentially within the central nervous system yielding distinct propagation patterns. We provide here the proof-of-concept that the conformation adopted by alpha-synuclein assemblies determines their ability to amplify and propagate in the brain in vivo. Our observations support the view that alpha-synuclein polymorphs may underlie different propagation patterns within human brains.

## Introduction

Synucleinopathies are a group of neurodegenerative diseases characterized by the progressive accumulation of abnormal proteinaceous aggregates in the central nervous system [25]. These inclusions are enriched in alpha-synuclein (α-syn), have different appearances inside cells and develop in selective brain regions and peripheral nervous tissues depending on the disease. In Parkinson’s disease (PD) and Dementia with Lewy bodies (DLB), the inclusions are predominantly neuronal and are called Lewy bodies and Lewy neurites [24, 59]. In contrast, in Multiple System Atrophy (MSA), the inclusions are mainly localized within oligodendrocytes and Schwann cells [43, 63]. In all the synucleinopathies, α-syn inclusions progressively involve more areas of the nervous system. The gradual increase in affected cells appears to follow patterns consistent with α-syn inclusions spreading along neuronal connections. Notably, different regions of the central nervous system are affected in distinct synucleinopathies. Inclusions in both PD and DLB progressively engage olfactory regions, brainstem, limbic regions, and finally the neocortex [3, 1, 6, 7, 12, 32]. However, PD pathology is believed to affect predominantly the brainstem while in DLB the limbic system is more involved at an earlier stage. Furthermore, in PD, but not in DLB, synucleinopathy is considered to affect the dorsal motor nucleus of the vagus at a very early stage [7]. In MSA, synucleinopathy occurs notably in the striatum, basal ganglia, pontine nuclei, cerebellum inferior olives and spinal cord [24, 65], and although not studied extensively, it has been suggested that the two main MSA subtypes MSA-P and MSA-C exhibit stereotypic patterns of rostro-caudal spread of α-syn inclusions [8, 30]. Finally, in pure autonomic failure, α-syn inclusions are localized in sympathetic nervous system [2].

It has been proposed that α-syn assemblies propagate in a prion-like fashion in the brain, a mechanism that could at least partly account for pathology progression according to stereotypic patterns in the different synucleinopathies. The prion-like behavior of α-syn assemblies has been studied in vitro and in vivo in animal models, and includes three distinct steps: the uptake of α-syn seeds by cells, the seeding of their cellular counterpart, and the traffic of aggregated α-syn within the neuron and to distant brain regions [21, 29, 40]. We, and others, have shown that α-syn pathology can be transmitted by inoculation into the brain or in peripheral organs of healthy wild-type (WT) animals of recombinant α-syn aggregates, or brain extracts from patients [11, 17, 52, 54, 38, 56, 57, 60, 62]. We have demonstrated widespread propagation of inclusions through the neuronal network upon injection of recombinant α-syn fibrils in the olfactory bulb (OB) of WT mice [42, 54, 56]. The inclusions developed in the brain following a pattern of propagation reminiscent of the early pathology in PD and DLB. It is unclear, however, why misfolded α-syn proteins trigger different types of pathologies in the different forms of synucleinopathy. Recently, we demonstrated that recombinant α-syn assembles in vitro into different fibrillar polymorphs that exhibit distinct biochemical, structural and physical characteristics [5, 37]. When incubated with monomeric α-syn or applied to cell cultures, these polymorphs seeded the aggregation of non-pathogenic α-syn and imprinted their distinct structures and biochemical characteristics to the recruited α-syn [5, 18, 19, 61]. When injected in vivo, the two polymorphs behaved as distinct strains, they promoted α-syn inclusions formation but triggered different types of neuropathology and behavioral changes [47]. Others have shown that MSA or PD brain extracts also act like strains in vitro [66] and in vivo depending on both the seed conformation and the intracellular environment [48]. We proposed that different α-syn strains contribute to the development of the known variety of synucleinopathies [41, 46] and that different polymorphs seed and spread to different degrees. To further explore this hypothesis, we generated and characterized five different α-syn fibrillar polymorphs. As the olfactory system is involved early in PD and DLB, and propagation of pathology through this network can be mapped [56, 54], we injected the five α-syn strains into the OB of WT mice and followed longitudinally and spatially pathology hallmarks for up to 6 months post injection (MPI). We show here that the different strains seed and propagate pathology throughout the olfactory network within the brain to different extents. We demonstrate strain-dependent inclusions formation in neurites or cell bodies.

## Materials and methods

### Study design

The goals of our study were to assess the pathological effects of different α-syn fibrillar strains in vivo and define their conformation-dependent seeding and propagation propensities following injection into the OB of WT mice. To this aim, we chose to work with in vitro generated assemblies, for which we can control the purity and the homogeneity of each strain. Our methods and our thorough quality controls guarantee that the differences between our wild type human α-syn strains reside uniquely in their intrinsic conformation/structure.

### Preparation of assemblies

Recombinant WT full length human α-syn or WT human C-terminal truncated (110) α-syn was expressed in *Escherichia coli* BL21 (DE3) (Stratagene, La Jolla, CA, USA) and purified as previously described [5, 18, 20, 23, 27, 37]. At the end of purification, we determined the concentration of α-syn by spectrophotometry at 280 nm using an extinction coefficient of 5960 M^− 1^ cm^− 1^ for WT human full length α-syn or 1490 M^− 1^ cm^− 1^ for C-terminal truncated α-syn. α-Syn (in 50 mM Tris-HCl, pH 7.5, 300 mM KCl) was then filtered through sterile 0.22 μm filters, aliquoted and stored at − 80 °C.

Monomeric α-syn (used as control here) was dialyzed against phosphate buffer saline (PBS), frozen in liquid nitrogen and stored at − 80 °C.

Using Pierce LAL Chromogenic Endotoxin Quantification kit (Thermo Fisher Scientific, #88282), we performed endotoxin detection as described previously [28, 47] and controlled that endotoxin levels were below 0.02 endotoxin units/μg.

We produced five different fibrillar α-syn polymorphs, including four different polymorphs of WT full length human α-syn assemblies, as described previously [5, 27, 37], and one strain of WT C-terminal truncated (aa 1–110) α-syn fibrils. To produce these different fibrillar polymorphs, α-syn was dialyzed against different buffers (500 μL against 4 L) and then incubated under continuous shaking (600 r.p.m.) at 37 °C in an Eppendorf thermomixer for 5 to 10 days depending on the fibrillar polymorph. For the polymorph fibrils, monomeric α-syn was incubated in 50 mM TrisHCl pH 7.5, 150 mM KCl buffer. For the polymorph ribbons, we dialyzed monomeric α-syn against 5 mM Tris-HCl pH 7.5 at 4 °C for 16 h prior to incubation. For the polymorph fibrils-65 (F-65), monomeric α-syn was dialyzed overnight at 4 °C against 50 mM MES pH 6.5, 150 mM NaCl. For the polymorph fibrils-91 (F-91), monomeric α-syn was dialyzed overnight at 4 °C against 25 mM Na_2_PO4 pH 9.1. Finally, for the strain fibrils-110 (F-110), C-terminally truncated α-syn was incubated in 40 mM TrisHCl pH 7.5, 150 mM KCl.

We monitored assemblies by measuring thioflavin T fluorescence in presence of 10 μM Thioflavin T (by spectrofluorimetry; excitation at 440 nm, emission at 440 and 480 nm). The fibrillar polymorphs were then centrifuged at 35000 g to eliminate remaining monomeric α-syn once assembly reaction reached steady state. We collected the supernatant and measured the concentration of monomeric α-syn (non-assembled) spectrophotometrically. The pelleted fibrillar polymorphs were then resuspended into sterile PBS to reach a final concentration of 350 μM (5 μg/μL) or 138 μM (2 μg/μL), then submitted to powerful sonication to fragment the assemblies into smaller fibrils using a sonotrode (sonication for 20 min, 0.5 s pulses; Sonicator UIS250V, equipped with VialTweeter, Hielscher Ultrasound Technology, Germany). Assemblies were then aliquoted and stored at − 80 °C (fibrils) or RT (other strains) for use within 10 days. The sonication was performed before aliquoting and freezing to ensure homogeneity between aliquots.

### Quality control of assemblies

#### Transmission electron microscopy (TEM)

We verified the nature of the α-syn assemblies by TEM after absorption onto carbon-coated grids using negative staining with 1% uranyl acetate (Jeol 1400 TEM; Gatan Orius CCD camera) (Additional file 1). The average apparent molecular weight of the fragmented fibrillar polymorphs we used throughout this study was assessed by analytical ultracentrifugation as in [28].

#### Limited proteolytic digestion

We performed proteinase-K limited digestion to finger print the different fibrillar polymorphs. Samples from the different assemblies were diluted to 100 μM in PBS and incubated at 37 °C with proteinase K (3.8 μg/mL). At different time intervals, samples were withdrawn from the degradation reaction and supplemented with protease inhibitor phenylmethanesulfonyl fluoride (PMSF) (to a final concentration of 3.3 μM). Samples were then frozen in liquid nitrogen and dehydrated. Fibrils were then disassembled by treatment with 100% hexafluoro-2-propanol (HFIP) for 1 h. Samples were then air-dried, dissolved in 15 μL of Laemmli sample buffer and denatured for 5 min at 80 °C. Samples were then analyzed on Tris-Glycine-SDS-polyacrylamide (15%) gel (SDS-PAGE), stained by Coomassie blue and imaged using a ChemiDocTM MP (BioRad) (Additional file 1).

#### Animals

C57Bl/6 J female mice were purchased from the Jackson Laboratories (USA) at the age of 7 weeks and were housed five per cage under 12 h light/12 h dark cycle with constant access to water and food. Mice were housed and handled in accordance with the Guide for the Care and Use of Laboratory Animals (US National Institutes of Health) and all procedures were approved by the Van Andel Institute’s IACUC (AUP 14–01-001 and AUP 16–02-033).

#### Stereotactic injections

Mice were injected at the age of 8–9 weeks (5 animals per group, except for monomer injected group where *n* = 3–4). On the day of use, the fibrils-strain (stored at − 80 °C) was thawed by incubation for 3 min in a water bath at 37 °C. Other strains were not frozen and stored at room temperature. Before injection, the assemblies stored at room temperature were gently sonicated for 5 min in a water bath ultrasonic cleaner (Sentry Digital Ultrasonic Cleaner, Cell Point Scientific, Gaithersburg, MD, USA) at room temperature to disperse the assemblies homogeneously.

We injected different α-syn assemblies (Fibrils, Ribbons, F-65, F-91, F-110) (0.8 μL per injection, 2 μg/uL of assembled α-syn in sterile phosphate buffer saline) unilaterally in the OB by stereotactic surgery following the procedure described previously [54, 55, 56]. Briefly, mice were anesthetized under a mixture of isoflurane/oxygen, and were injected using a thin glass capillary attached to a 10 μL Hamilton syringe (coordinates: AP: + 5.4 mm; L: − 0,75 mm, DV: − 1 mm relative to bregma and dural surface) at a constant rate of 0.2 μL/min. The injection coordinates and volume injected were set after extensive pilot testing to minimize the amount of fibrils reaching the ependymal and subependymal layer of the OB and to avoid passive diffusion to neighboring brain regions [55]. The capillary was kept in place for 3 min after the end of the injection. One mouse injected with strain P-65 was found dead for unknown reason 5 MPI and was excluded from the study.

#### Preparation of the tissue

At 3- or 6-MPI, mice were deeply anesthetized by sodium pentobarbital intra-peritoneal injection and perfused transcardially with 0.9% saline, followed by cold 4% paraformaldehyde (PFA). We collected the brains and post-fixed them for 24 h in 4% PFA at 4 °C and then stored them in 30% sucrose solution until sectioning. The brains were then sectioned on a freezing microtome into 6 series of 40 μm free floating sections.

#### Immunohistochemistry

To detect α-syn inclusions in mouse brain tissue, we stained one series of coronal sections (210 μm interval between consecutive sections) from each animal, 3–5 animal per group (monomers, Fibrils, Ribbons, F-65, F-91, F-110) and per delay (3- and 6-MPI) with an antibody directed against α-syn phosphorylated on serine 129 (pser129) and biotinylated rabbit antisera (Please refer to Additional file 2 for antibodies’ concentration and references) [54, 56]. We then used a standard peroxidase-based method to detect the antibody with 3, 3′-diaminobenzidine (DAB, Vectastain ABC HRP kit and DAB Peroxidase HRP kit, Vector Laboratories).

Sections were mounted on slides and gradually dehydrated before slides were coverslipped with Cytoseal 60 mounting medium (Thermo Fisher Scientific).

**Fig. 1:**
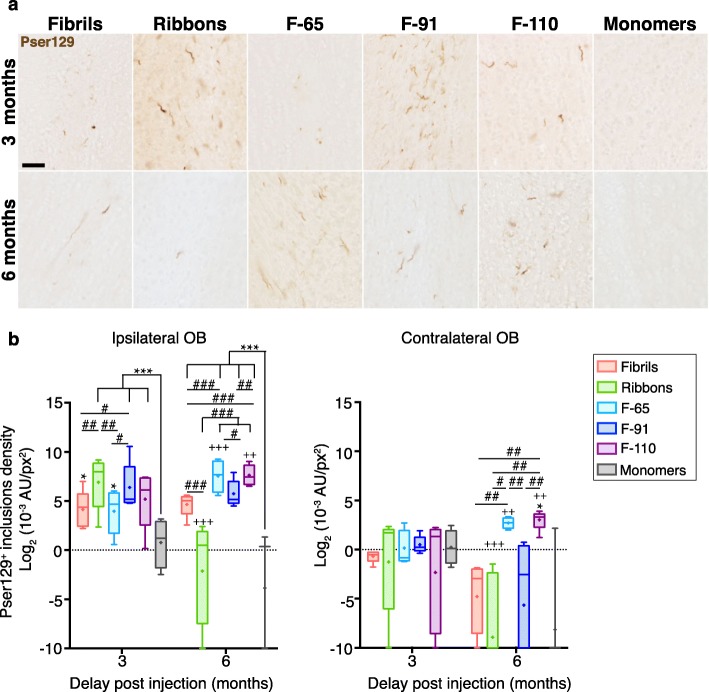
Different α-syn strains induce pser129 inclusions in the olfactory bulb with different seeding efficiencies. **a** α-Syn inclusions in the injected olfactory bulb (OB) at 3- and 6-months post injection (MPI), detected by an antibody anti-α-syn phosphorylated on serine 129 (pser129). **b** α-Syn inclusions measured by densitometry of the pser129 staining in ipsilateral and contralateral OB, 3- and 6-MPI. Immunostaining for pser129 was performed in eight independent histochemical experiments. Densitometry performed on 3–5 animals per group (3 MPI: monomers, *n* = 4; Fibrils, *n* = 5; Ribbons, *n* = 5; F-65, *n* = 5; F-91, *n* = 5; F-110, *n* = 5; 6 MPI: monomers, *n* = 3; Fibrils, *n* = 5; Ribbons, *n* = 5; F-65, *n* = 4; F-91, *n* = 5; F-110, *n* = 5). Statistical analyses were performed by linear mixed-effect model and included 3 factors (strains, ipsilateral / contralateral sides, and delay post injection). Data are presented after log_2_-transformation. Boxes represent the 25th to 75th percentiles. The median and the mean are represented in each box by the line and the cross respectively. Error bars correspond to the minimal and maximal values measured. * comparison monomers to strains within same group of age and side; # comparison between strains within same group of age and side; + comparison between 3 and 6 months, same injectate, same side. No significant interaction between time and side. Statistics are available in Additional file 5. Scale bar: 25 μm

**Fig. 2:**
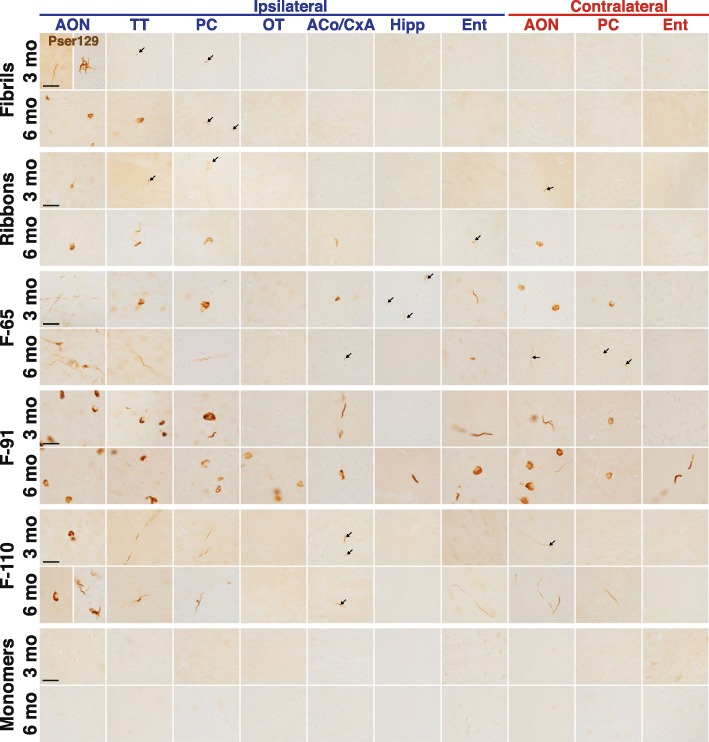
Strain-induced synucleinopathy propagates to interconnected brain regions with different spatial pattern depending on the strain. α-Syn inclusions detected in some ipsilateral (legend in blue) and contralateral (legend in red) brain regions at 3- and 6-MPI. α-Syn inclusions were detected by an antibody directed against α-syn phosphorylated on serine 129 (pser129). Immunostaining for pser129 was performed in eight independent histochemical experiments (3 MPI: monomers, *n* = 4; Fibrils, *n* = 5; Ribbons, *n* = 5; F-65, *n* = 5; F-91, *n* = 5; F-110, *n* = 5; 6 MPI: monomers, *n* = 3; Fibrils, *n* = 5; Ribbons, *n* = 5; F-65, *n* = 4; F-91, *n* = 5; F-110, *n* = 5). A list of brain structure abbreviations is provided as Additional file 4. Scale bar: 25 μm

Representative images of pser129 staining in Figs. 1 and 2 were acquired at 63x magnification on a Leica DM6000 microscope and examples of low power images acquired at 5x magnification are presented in Additional file 3.

#### Scoring of pser129 pathology (based on DAB staining) and generation of heat maps

All experiments were performed blinded. A second individual assigned new names to stained slides prior to analysis. To investigate pser129 pathology on the blind coded slides, we noted the presence of pser129-positive inclusions in one series of coronal sections per animal, as described previously [54]. We examined the entire surface of every section at 20x magnification on a Nikon Eclipse Ni-U microscope and defined a score for each brain region depending on the abundance of pser129 somatic and neurite pathology observed. Scores ranged from 0 to 4 corresponding to “0= no inclusions”, “1= sparse inclusions”, “2= mild burden”,“3 = dense burden”, and “4= very dense”. We calculated the average score per brain region within the same experimental group and represented the results on heat maps (Fig. 4) generated on the software R v3.2.1 [50] (https://cran.R-project.org). A list of abbreviations used for brain regions in the heat maps is available as Additional file 4.

#### Densitometry of pser129 inclusions

We investigated the extent of pser129 inclusions in the OB, the anterior olfactory nucleus (AON), the piriform cortex (PC) and the entorhinal cortex (Ent) of mice injected with different strains 3- and 6-MPI by densitometry as described previously [54]. Briefly, we acquired photomicrographs from blind-coded slides at 20x magnification using the same exposure parameters on three consecutive sections distanced by 420 μm intervals for OB and AON; 840 μm intervals for the Ent and eight consecutive sections distanced by 630 μm intervals for the PC.

The images were then analyzed in ImageJ64 [51] as detailed previously [54] to obtain the area of the region of interest (px^2^) and the mean grey value (A.U.) of the pser129-positive area. We determined the average grey value per square pixels for each brain region and animal and calculated the average grey value per square pixels for each brain region and experimental group.

Because densitometry data is more similar to count data than Gaussian (number of pixels/area squared), each brain region was analyzed individually using a negative binomial mixed-effects model with random intercepts for each individual, via R v 3.4.3 (https://cran.r-project.org/). The interactions: *treatment-group x time* and *side x time* were assessed individually for each region; this interaction was dropped from models where it was not significant at alpha = 0.1. Benjamini-Hochberg adjusted linear contrasts were used to test specific hypotheses via the R package ‘emmeans’ [34] (https://CRAN.R-project.org/package=emmeans). We have performed power calculations for similar data for an earlier study [54] and have found typically 4–6 animals/group/timepoint yields > 80% power for what are expected to be large effects (> 2 fold differences). A power calculation done after this experiment that made only assumptions about the number of comparisons being made in these data and their distributions, estimated that 5 animals included per group and timepoint would have > 80% power to detect ~ 2.2 fold changes in the density measured as A.U/px^2^. Thus, although these data did not have their own power calculation, and acknowledging that a post-hoc calculation no longer has any meaning to the data that were already collected, the densitometry analysis was still designed with reasonable power to detect large, but not unattainable changes in densitometry.

The anterior and posterior PC were first analyzed separately. There was very little evidence to suggest the densitometry data from the anterior and posterior PC differed via significance testing and qualitative inspection of the distribution. We thus pulled together anterior and posterior PC data for statistical analysis. Statistical analysis data are available in Additional file 5. Large differences were observed between some groups, and to represent them better on graphs, individual values expressed as 10^− 3^ A.U/px^2^ were log_2_-transformed before plotting. As the density of pSer129 inclusions detected was equal to 0 in some individuals, we calculated log_2_(individual value + 0.0001) to avoid log_2_ transformation of 0 which does not exist. Negative values after log_2_ transformation correspond to values ranging between 0 and 1, related to very low basal/background signal detected. Boxes in the box plots represent the 25th to 75th percentiles, the median and the mean are represented in each box by the line and the cross respectively. Error bars correspond to the minimal and maximal values measured. Graphs were designed in Prism 6.0, GraphPad.

#### Immunofluorescence

We stained the sections with primary antibodies and appropriate secondary antibodies listed in the Additional file 2.

For Thioflavin S/NeuN staining, NeuN was detected in sections (one half series per animal, *n* = 3–4 per group at 3 MPI, randomly selected) using the antibodies listed in Additional file 2; DAPI (1:10000) was added to the secondary antibody solution. After washing, the sections were mounted on glass slides, dried, rehydrated and incubated for 8 min at RT with 0.05% filtered thioflavin S (ThS) diluted in distilled water. Sections were dehydrated in 80, 95% and then 95% ethanol and treated with TrueBlack Lipofuscin Autofluorescence Quencher (Biotium, Fremont, CA, USA) at 1:20 concentration in 70% ethanol for 30 s, washed in PBS and mounted with EverBrite Mounting medium (Biotium). We stained 3–5 animals’ brains per group from the 3-MPI delay and blind-coded the slides for confocal analysis. We acquired multichannel confocal stacks of ThS-positive inclusions on an inverted Nikon A1 plus-RSi laser scanning confocal microscope, using 403, 488, 561, 640 nm solid state lasers. Stacks were then processed on NIS Elements AR 4.00.08 software (Nikon) to apply a median filter (kernel 3) (Fig. 6a).

For triple staining with NeuN, Pser129 and olig2, we used a conjugated olig2-AF488 antibody since antibodies to both olig2 and Pser129 were made in rabbit. Sections of OB and AON (one series per animal, for each animal at 6 MPI, 3–5 animals per group) were pretreated in Tris-EDTA pH 9.0 antigen retrieval solution 1x (10x Tris-EDTA retrieval buffer pH 9.0, K043, Diagnostic Biosystems Pleasanton, CA, USA) for 30 min at 90 °C; then incubated with primary antibodies directed against NeuN and anti-pser129 overnight at 4 °C (blocking in normal goat serum); then with secondary antibodies conjugated to Alexa633 and to Alexa568 for 2 h. Sections were then blocked with 2% rabbit sera with extensive washing between each step. We then incubated the sections 48 h at 4 °C with olig2-AF488 antibody (extensive testing demonstrated no cross-binding between olig2 and pser129, data not shown).

Blind-coded sections were then imaged on an inverted laser scanning confocal microscope (Leica SP8 X equipped with white light laser 2 and Diode 405 nm; 63x-oil immersion objective) using sequential acquisition between frames (excitation 633, 568, 488 and then 403) with a step size of 0.3 μm between stacks.

In an attempt to perform quantifications of pser129 inclusions in NeuN- or Olig2-positive cells, we acquired Z stacks from 20 somatic pser129-positive inclusions (in proximity to a DAPI-positive nucleus) per animal throughout the AON. In some groups (fibrils, F-65 and F-110), the number of somatic inclusions was too low to reach this number, so no statistical analysis of the results could be performed. We then analyzed the stacks after applying a median filter (kernel 3) using the software LAS-X to assess whether inclusions are localized within NeuN^+^ or Olig2^+^ cells. Orthogonal and maximal intensity projections were generated on LAS-X (Fig. 6b, c).

#### Assessment of microglial morphology

Morphological analysis of labeled microglia (*n* = 4 animals per strain) was performed for changes in area/perimeter (hydraulic radius). We acquired fluorescent images of Iba-1-stained ipsilateral OB sections at 60x magnification using a Nikon A1plus-RSi laser scanning confocal microscope system. Nine images were analyzed per animal (three sites per section, three sections per mouse). Microglia segmentation of confocal stacks was performed using Imaris 3.0 (Bitplane) using the surfaces tool on the green channel. Files containing segmented microglia were then exported as TIFF files and were processed by a custom MATLAB (Mathworks) script based on what was previously described [22]. The calculated ratio of area:perimeter (hydraulic radius) was used as a measure for microglial activation; activated microglia are amoeboid in shape and therefore have a larger index score. Differences in means between the groups were analyzed using a one-way ANOVA test by using GraphPad Prism software.

## Results

### Characterization of α-syn fibrillar polymorphs

We hypothesized that the injection of different α-syn fibrillar polymorphs into the OB of WT mice would trigger distinct α-syn pathology and propagation patterns because of differential α-syn strains seeding propensity and spread. In order to test this hypothesis, we generated five different α-syn fibrillar polymorphs. We assessed strains preparations characteristics and homogeneity before use. First, we confirmed by TEM that the different α-syn fibrillar polymorphs were morphologically distinct, highly homogenous, and that their morphology observed by TEM is in agreement with our earlier work (Additional file 1) [5, 16, 26, 37]. Then we finger printed the different α-syn fibrillar polymorphs using their proteinase K degradation profile on SDS-PAGE (15%) following Coomassie blue staining. The rationale for this approach is that proteinase K will access its cleavage sites in α-syn when they are exposed to the solvent. As the latter is dependent on the structure of the fibrillar polymorphs, exposed residues and buried sequences will vary between polymorphs allowing us to verify that α-syn is in different conformations in distinct polymorphs. As expected, the limited proteolysis profiles were characteristic of each strain, confirming that they were pure and exhibiting distinct structural characteristics (Additional file 1), consistent with our previously published work.

Once characterized, the distinct fibrillar polymorphs were injected unilaterally into the OB of WT mice, using a protocol described previously [54, 55, 56].

### α-Syn pathology at the injection site

As in our earlier published work [54, 56], we assessed α-syn pathology at the injection site (OB) at 3 MPI using an antibody directed against α-syn phosphorylated on serine 129 (pser129), commonly used as a marker for α-syn inclusions [3, 53]. As expected, all the strains induced the formation of pser129 inclusions in the ipsilateral OB at 3 MPI (Fig. 1a) and inclusions were mostly in neurites. Control injections of α-syn monomers did not induce pser129 pathology in the OB. The pser129-positive inclusions were still present in the OB at 6 MPI for all the groups.

To further investigate the seeding efficiency of different strains at the injection site, we measured the density of pser129-positive inclusions at 3- and 6-MPI in the OB. At 3 MPI, the mice injected with the five α-syn strains exhibited a significantly higher pser129 signal compared to mice injected with α-syn monomers (Fig. 1b; statistics available in Additional file 5). Animals injected with Ribbons or F-91 assemblies displayed a significantly 2- to 10-fold higher density of inclusions than mice injected with the strains Fibrils or F-65, indicating lower seeding efficiency for the latter. Compared to the 3-month time point, at 6 MPI the density of F-110- and F-65-induced inclusions had increased significantly (+ 185% and + 850%, respectively), while the density of Ribbons-induced inclusions decreased drastically (− 99.2%). Finally, the density of Fibrils- and F-91-induced inclusions did not change significantly over time. Our results suggest that F-110 and F-65-induced inclusions persisted and seeded endogenous α-syn aggregation further from the injection site at 6 MPI. In contrast, Ribbons-induced inclusions disappeared rapidly from the injection site. Finally, we observed differences in the density of pser129 inclusions between ipsilateral and contralateral brain regions for a given strain (for example, Fig. 5b, F-91 mice, mean density of inclusions at 3 MPI: Density in ipsi PC is 10-times higher than in contra PC; at 6 MPI, density in ipsi PC is increased by 10-fold compared to ipsi PC at 3 MPI, and is also about 10-times higher than the density in contra PC at 6 MPI) but we found no significant interaction between time and side of the brain. Thus, there is not enough evidence suggesting that pathology triggered by a given strain in the two sides of the brain evolved differently over time.

### The five strains triggered propagation of pathology in the brain

To investigate the propagation of α-syn pathology following the injection of the different strains, we performed pser129 immunostaining on the entire brain of mice at 3- and 6-MPI.

We detected pser129-positive inclusions in regions distant from the OB, in each strain-injected group. The extent of pathology propagation differed from one strain to another. Fibrils-induced inclusions propagated to a comparatively small number of brain regions close to the OB, while other strains propagated to many more brain regions (proximal and distant) (High magnification micrographs in Fig. 2; examples of low power images in Additional file 3). After injection of strain F-110, we observed mostly inclusions in neurites in the brain regions distant from the OB. By contrast, in mice injected with fibrils, ribbons and F-65 strains there were inclusions both in neurites and cell bodies, and animals injected with strain F-91 had particularly numerous somatic inclusions.

### Anatomical pattern of α-syn pathology propagation following strains injection

**Fig. 3:**
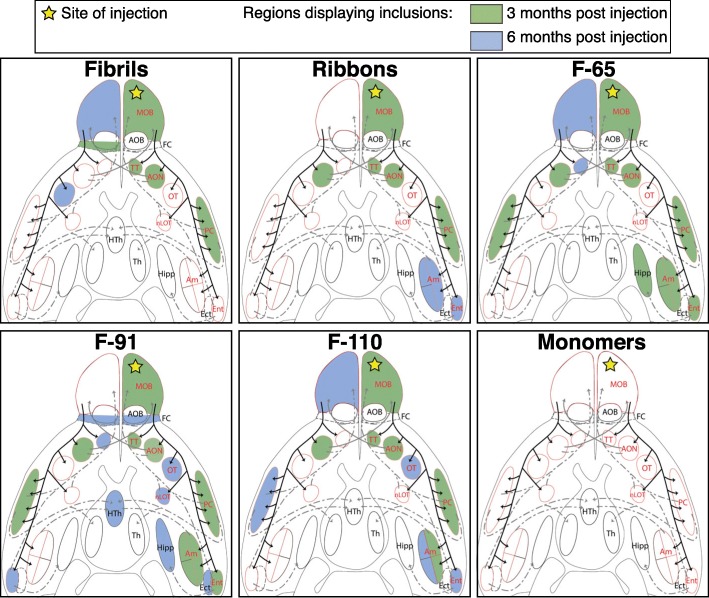
α-Syn pathology spreading pattern depends on the strain injected: spreading in the olfactory system. Schematic of ventral brain regions representing the main primary (solid black lines) and secondary connections from the OB or to the OB (dashed or solid grey lines). Brain structures with red outline correspond to brain regions that are directly connected to the OB. The yellow star indicates the site of injection. Areas colored in green and blue represent major regions displaying pser129 inclusions at 3- and 6-months respectively (non-exhaustive representation). A list of brain structure abbreviations is provided as Additional file 4

**Fig. 4:**
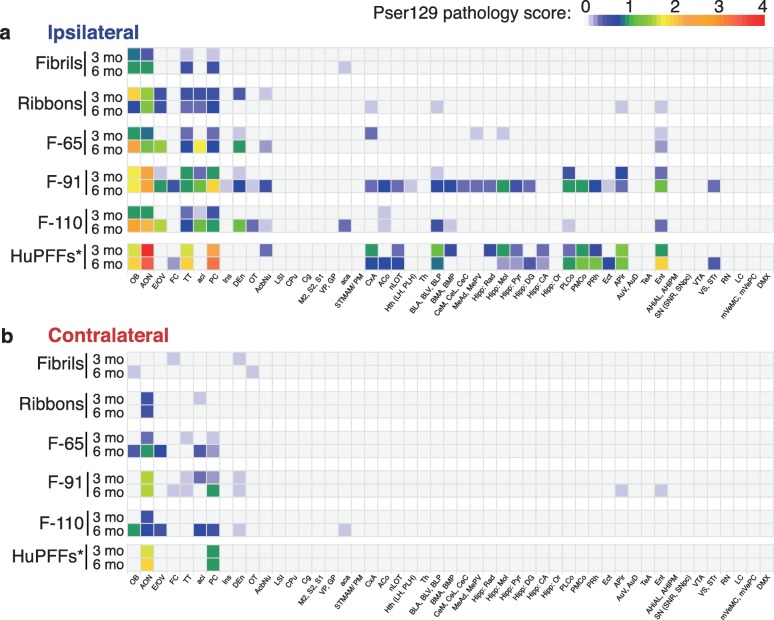
α-Syn pathology spreading pattern depends on the strain injected: exhaustive analysis. Heatmap representing the severity of α-syn pathology (pser129 inclusions) in numerous brain regions, ipsilateral (**a**) (legend in blue) and contralateral (**b**) (legend in red) to the injection site, 3- and 6-months post injections of different strains (exhaustive representation) or of human α-syn preformed fibrils (huPFFs; from the laboratory of V.M-Y Lee). Data for the huPFFs were obtained from previous experiments, published in [54, 56]. The colors code for the mean score of each group per designated brain region and the scoring was performed on a scale from 0 to 4 in 3–6 animals per group (3 MPI: monomers, *n* = 4; Fibrils, *n* = 5; Ribbons, *n* = 5; F-65, *n* = 5; F-91, *n* = 5; F-110, *n* = 5; huPFFs, *n* = 4; 6 MPI: monomers, *n* = 3; Fibrils, *n* = 5; Ribbons, *n* = 5; F-65, *n* = 4; F-91, *n* = 5; F-110, *n* = 5; huPFFs, *n* = 4). A list of brain structures abbreviations is provided as Additional file 4. * The huPFFs were injected at a higher concentration (5 μg/μL) than the strains (fibrils, ribbons, F-65, F-91 and F-110) and the control (monomers) used in this work (2 μg/μL); the volume injected remained the same (0.8 μL, 4 μg of huPFFs versus 1.6 μg of each strain per OB)

To neuroanatomically map the propagation of α-syn pathology, we plotted its distribution on a schematic drawing depicting the ventral side of the mouse brain, where we highlighted the main brain regions connected to the OB (Fig. 3). The yellow star represents the injection site, and brain regions that exhibited inclusions at 3- and 6-MPI are shaded green and blue, respectively (Fig. 3). In addition, in order to visualize pathology spreading and to assess it semi-quantitatively we scored pser129 inclusions abundance (cell bodies and processes) on a scale from 0 to 4 in the entire brain of each mouse. We then calculated the mean score for each brain region, group and timepoint and depicted the data in heatmaps (Fig. 4).

### Injections of α-syn fibrils

At 3- and 6- MPI, we did not detect any pser129 inclusions in any brain region of control mice injected with monomers (score = 0) (Figs. 3 and 4). By contrast, at 3 MPI, Fibrils triggered inclusions in brain regions that are directly connected to the OB (tenia tecta (TT), AON, PC notably [15]). However, overall the pathology was mild to moderate (score between 0 and 2) (Fig. 4) and restricted to rostral brain regions (Fig. 3). At 6 months after Fibrils injection, we found additional pathology in the contralateral OB and olfactory tubercle (OT), localized one distant synaptic relay from the ipsilateral regions affected at 3 months. Once again, α-syn pathology remained mild and sparse (mean score ≤ 1; Fig. 4) and had not spread to distant connected brain regions (Figs. 3 and 4).

### Injections of α-syn ribbons

At 3 MPI of α-syn Ribbons, pathology was present in the same olfactory regions ipsilateral to the injection as in the α-syn Fibrils-injected group. We also found inclusions in the contralateral AON, which has efferent projections going to the OB that was injected (Fig. 3). The OB exhibited moderate pathology (score > 1 and ≤ 2) and the ipsilateral AON displayed mild pathology (score > 0 and ≤ 1). Aside from these two regions, all the other affected regions at most displayed occasional sparse pathology (score ≤ 1) (Fig. 4). At 6 MPI, sparse inclusions (score ≤ 1) were present in ipsilateral olfactory regions directly connected to the OB by both centripetal and centrifugal connections [9, 15], but located distant from the injection site, namely basolateral amygdala (BLA), cortex-amygdala transition area (CxA) and the entorhinal cortex (Ent) (Figs. 3 and 4). In summary, pathology induced by injection of α-syn Ribbons was primarily restricted to the olfactory system and regions directly connected with the injected OB.

### Injections of α-syn strain F-65

At 3 MPI, the F-65 α-syn strain induced more widespread, albeit sparse (score ≤ 1), pathology than the two strains described above. Inclusions were located in most of the olfactory regions directly connected to the injected OB (ipsilateral TT, PC, CxA, Ent, ipsi- and contralateral AON), and also in regions localized one synapse away from first order olfactory regions (ipsilateral Medial nucleus of the amygdala (MeAD, MePV), ipsilateral hippocampus (Hipp), contralateral PC [10, 15]; (Figs. 3 and 4).

At 6 MPI, additional pathology was present in the contralateral TT and OB, two distant synaptic relays from the injected OB. Sparse pathology seen in some brain regions (e.g. CxA, MeAD, MePV, Hipp) at 3 months was no longer present at 6 months. Nevertheless, the pathology was greater (score = 1–3) in the most rostral olfactory regions at 6 compared to 3 MPI (Fig. 4).

### Injections of α-syn strain F-91

The pattern of propagation of pathology after injection of the F-91 α-syn strain was similar to that seen after injection of strain F-65 (Figs. 3 and 4). At 3 months, F-91-triggered inclusions were present in all the olfactory regions that are directly connected to the injected OB except in the OT and the nucleus of the lateral olfactory tract (nLOT). In addition, inclusions also reached the ipsilateral BLA (that projects onto the Ent) and the contralateral PC (receiving connections from the contralateral AON), both localized at least one distant synaptic relay from primary olfactory regions (Figs. 3 and 4). At 6 MPI, pathology reached many additional brain regions, including both regions that receive direct projections from the OB and several distant brain regions that are indirectly connected to the injection site. At this time point, pathology ranged from sparse/mild to substantial (score rising from 0 to 1–3) depending on the target region (Fig. 4).

In summary, the strain F-91 appeared to be the most efficient strain at inducing pathology propagation in terms of spatial extent, and possibly in terms of pathology severity (investigated further in the next section).

### Injections of α-syn strain F-110

Finally, injection of the α-syn strain F-110 triggered the formation of α-syn inclusions in many brain regions. At 3 MPI, sparse to mild inclusions were present in most of the olfactory regions directly connected to the OB (TT, AON, PC, ACo and contralateral AON). At 6 MPI, pser129 α-syn was also detected in the ipsilateral OT, Ent and BLA, MeA, PLCo, and had reached the contralateral OB and PC (both connected to the contralateral AON) (Fig. 3). In the proximal olfactory regions, the pathology had progressed from mild at 3 months to substantial at 6 MPI (Fig. 4). In a direct comparison, strain F-110 propagated efficiently although not to the same extent than strains F-91 and F-65.

In conclusion, Fibrils- and Ribbons-induced pathology appeared to propagate the least, F-91-induced pathology propagated the most, and strains F-65 and F-110 led to an intermediate figure where propagation was slightly faster for F-65 (reaching Ent and the entire Amygdala already by 3 months). We also observed that strain F-91 induced substantial pathology in the AON at 3 MPI, and F-91- and F-110-induced pathology reached substantial levels in the OB and/or the AON at 6 MPI.

### Different α-syn strains induce different patterns of pathology

To define quantitively the inclusions induced by the different strains, we assessed pser129 inclusions density in key brain regions directly connected to the injection site.

First, we quantified staining in the contralateral OB at 3 and 6 MPI (Fig. 1b). The contralateral OB receives inputs from the ipsilateral AON and is connected to the contralateral AON. At 3 MPI, we did not detect inclusions in the contralateral OB (Figs. 3 and 4), and consistently, the densities of inclusions for each strain-injected group were not significantly different from the control (monomers) group (value between 0.65 × 10^− 3^ A.U/px^2^ and 2.18 × 10^− 3^ A.U/px^2^ and between − 0.621 and 1.124 after log_2_ transformation, respectively), corresponding to minimal background detection) (Fig. 1b). At 6 MPI, we detected a significant increase of pser129 pathology for F-65- and F-110- injected groups compared to 3 MPI (+ 239% and + 335% respectively). The density of inclusions in F-110-injected animals at 6 months was significantly higher from that of Fibrils-, Ribbons- and F-91-injected groups. In addition, the density of inclusions in the F-65-injected group was significantly greater than in the Fibrils-, Ribbons- and F-91-injected groups, but did not reach significance when compared to animals injected monomers (*p* = 0.054). In summary, only strains F-65 and F-110 triggered clear propagation to the contralateral OB at 6 MPI.

**Fig. 5:**
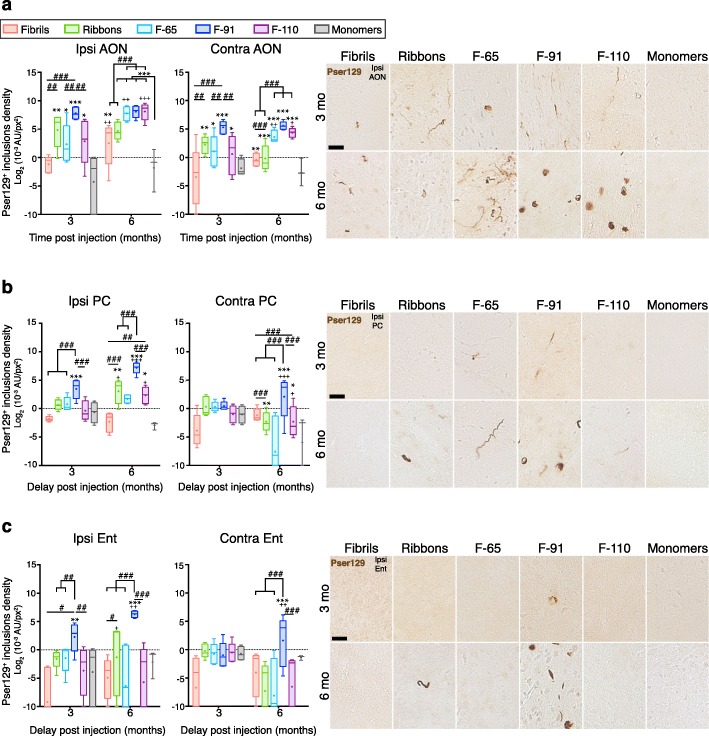
The density of α-syn inclusions in connected brain regions depends on the strain used. We measured the densitometry of pser129 staining in ipsilateral and contralateral AON (**a**), PC (**b**), Ent (**c**) at 3- and 6-MPI. Photomicrographs shown are representative of the average densitometry measured for each condition. Immunostaining for pser129 was performed in eight independent histochemical experiments. Densitometry performed on 3–5 animals per group (3 MPI: monomers, *n* = 4; Fibrils, *n* = 5; Ribbons, *n* = 5; F-65, *n* = 5; F-91, *n* = 5; F-110, *n* = 5; 6 MPI: monomers, *n* = 3; Fibrils, *n* = 5; Ribbons, *n* = 5; F-65, *n* = 4; F-91, *n* = 5; F-110, *n* = 5). Statistical analyses were performed by linear mixed-effect model and include three factors (strains, ipsilateral /contralateral sides, and delay post injection). Data are presented after log_2_-transformation. Boxes represent the 25th to 75th percentiles. The median and the mean are represented in each box by the line and the cross respectively. Error bars correspond to the minimal and maximal values measured. * comparison monomers to strains within same group of age and side; # comparison between strains within same group of age and side; + comparison between 3- and 6- MPI, same injectate, same side. Statistics are available in Additional file 5. Scale bar: 25 μm

Then, we assessed pser129 inclusions in the AON bilaterally. Despite the detection of sparse Fibrils-induced inclusions in the ipsilateral AON at 3 MPI, the density of inclusions was not statistically different from the control group (Fig. 5a). However, the densities of inclusions in the Ribbons-, F-65-, F-91- and F-110-injected groups were significantly higher that of the control group (monomers). Moreover, the F-91-induced inclusions were significantly greater (~ + 385% to + 760%) than Fibrils-, F-65- and F-110-induced inclusions indicating that the strain F-91 is the strain that propagated the most to the ipsilateral AON at this time point.

At 6 MPI, inclusions increased significantly in the ipsilateral AON of Fibrils- (~ + 3100%), F-65- (+ 450%) and F-110- (+ 963%) injected animals compared to the 3-months’ time point, while F-91 inclusions remained very dense (~ 329 × 10^− 3^ A.U/px^2^ corresponding to 8.362 after log_2_ transformation). The density of Ribbons-induced inclusions, however, decreased at 6 MPI (− 44%) (Fig. 5a).

Densitometry measurements in the contralateral AON revealed no inclusions in Fibrils-injected animals and significant inclusions load in Ribbons-, F-65-, F-91- and F-110-injected animals at 3 MPI, with F-91-induced inclusions surpassing the other strains by their density (+ 568% to + 773% higher than the other strains) (Fig. 5a). Hence, the results confirm the propagation of pathology to the contralateral AON we had observed previously (Figs. 3 and 4). At 6 MPI, the density of inclusions in the F-65- and F-110-injected groups increased significantly compared to the earlier timepoint (+ 83% and + 139%, respectively). Pathology density in the F-91-injected group did not change over time but remained high (53.5 × 10^− 3^ A.U/px^2^ corresponding to 5.741 after log_2_ transformation). In summary, the strain F-91 spread and triggered strong pathology in the contralateral AON. Comparatively, F-65 and F-110 induced milder pser129 pathology.

We also analyzed inclusion density in the PC, a structure that is directly connected to the OB. We first analyzed both the anterior and posterior PC since their connectivity to the OB is slightly different. The posterior PC receives projections only from mitral cells of the OB while the anterior PC receives afferences from both mitral and tufted cells. No statistical differences were observed between the anterior and posterior PC with regards to pathology density (Ipsilateral side: *p* = 0.58, 95% CI aPC vs pPC = [− 42%, 161%]; Contralateral: *p* = 0.1955, 95% CI aPC vs pPC = [− 20%, 186%]), so we pooled our data to analyze the whole PC at once.

In the ipsilateral PC, the density of pser129 inclusions in the F-91-injected group at 3 MPI was significantly higher than in other groups (+ 694% to + 1217%). Despite the observation of sparse inclusions in the ipsilateral PC in Fibrils-, Ribbons-, F-65- and F-110-injected animals at 3 months (Figs. 3 and 4), their densities were not high enough to be statistically different from the control group (Fig. 5b). At 6 MPI, the density of pser129 inclusions in the ipsilateral PC of Ribbons-, F-91-, and F-110-injected animals increased significantly (+ 893%, + 917% and + 437% respectively), the density of F-91-induced inclusions being far above the two other groups. Thus, the strain F-91 propagated the most to the ipsilateral PC and Ribbons- and F-110-inclusions became significant 3 months later than F-91-induced inclusions.

In the contralateral PC, we observed very sparse inclusions 3 months post injection of F-65 and F-91 strains, however, no significant differences could be detected when assessing the density of F-91-inclusions versus the control group (Fig. 5b). At 6 MPI, the density of inclusions increased significantly in the F-91-injected group (+ 731%) indicating efficient propagation to the contralateral PC.

Finally, we measured inclusion density in the Ent, the most caudally located olfactory structure that is directly connected to the OB. In the ipsilateral Ent, the sparse inclusions observed 3 months post F-65-injection and 6 months post F-110-injection (Figs. 3 and 4) were not registered by densitometry since we observed no significant difference compared to the control group (Fig. 5c). However, the density of F-91-induced inclusions was significantly elevated at 3 MPI and increased at 6 months (+ 780%). A significant density of Ribbons-induced inclusions was detected also in the ipsilateral Ent at 6 MPI indicating that Ribbons were efficient at propagating even though the density of inclusions remained low and decreased very quickly with time in the first regions affected (Fig. 1). In the contralateral Ent, no significant signal was observed at 3 MPI, consistent with the absence of inclusions in all groups (Figs. 3 and 4). Inclusions in the contralateral Ent 6 MPI were only observed in the F-91-injected group (Figs. 3 and 4), and as expected, only the F-91-injected mice exhibited high and significant pser129 density in the contralateral Ent (Fig. 5c; about 20 × 10^− 3^ A.U/px^2^ versus 0–0.2 × 10^− 3^ A.U/px^2^, corresponding to 4.32 and − 2.32 after log_2_ transformation).

Finally, we did not observe robust significant interaction between time and side for any strain. For this reason, we cannot conclude that pser129 pathology spreads differently over time on the two sides of the brain.

### Characterization of α-syn inclusions and their cellular predilection

We then defined which cell types the pser129 inclusions were present in, and what the morphological features of the pser129 inclusions were.

**Fig. 6:**
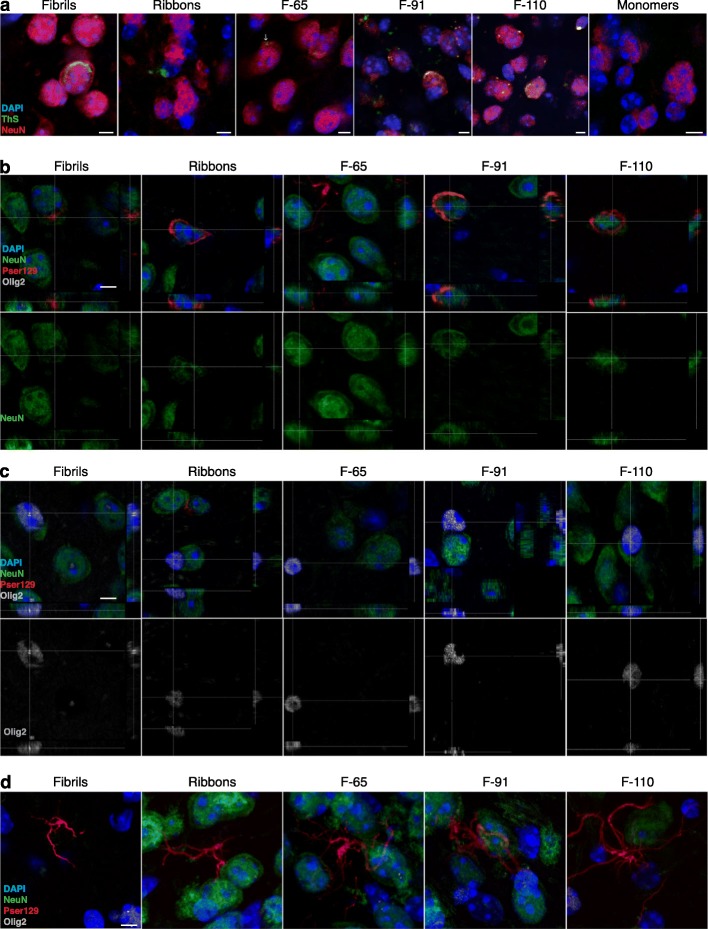
Inclusions are Thioflavin S positive and predominantly localized within neurons. **a** NeuN-positive cells (red), Thioflavin S (green) and DAPI-positive nuclei (blue) in the ipsilateral AON (monomers and all strains except ribbons) or OB (ribbons) at 3 MPI of the different strains or of the monomers. Photomicrographs were obtained from confocal stacks. The immunostaining was accomplished in two independent experiments, each on a half series of sections from 3 to 4 animals per group (3 MPI: monomers, *n* = 3; Fibrils, *n* = 4; Ribbons, *n* = 4; F-65, *n* = 4; F-91, *n* = 4; F-110, *n* = 4). Scale bars: 5 μm. **b**-**d** Staining of NeuN (green), Pser129 (red), Olig2 (white) and DAPI (blue) in the ipsilateral AON, at 6 MPI of the different strains. Orthogonal projections of Pser129+/NeuN+ cells (**b**) or olig2+ cells (**c**) and maximal projection images of pser129 processes-shaped inclusions (**d**) are obtained from confocal stacks. The immunodetection was performed in four independent experiments in one series of sections per animal, in 3–5 animals per group (6 MPI: monomers, *n* = 3; Fibrils, *n* = 5; Ribbons, *n* = 5; F-65, *n* = 4; F-91, *n* = 5; F-110, *n* = 5). Scale bar: 5 μm

To verify that the injection of α-syn fibrillar polymorphs triggered the formation of mature amyloid inclusions, we acquired confocal images in sections that we had first immunostained with an antibody to NeuN and then stained for amyloids using Thioflavin-S. We observed Thioflavin S-positive inclusions in each strain-injected group, while no Thioflavin-S signal was detected in the monomer-injected group, as expected (Fig. 6a).

Next, we analyzed cell-type specific markers to determine which cell types contained the pser129-immunoreactive inclusions in the ipsilateral AON of mice injected with different α-syn strains. We performed triple immunostaining for pser129 (red), NeuN (green), and Olig2 (white) to detect α-syn inclusions, neurons and oligodendrocytes, respectively (Fig. 6b-d). We analyzed these sections by confocal microscopy.

Most of the pser129-immunoreactive inclusions that were present in cell bodies were in NeuN-positive neurons while no somatic inclusions (Fig. 6b) were detected in olig2-positive oligodendrocytes (Fig. 6c). For Fibrils-injected animals, 100% of somatic inclusions were in NeuN-positive neurons. Because the number of inclusions that we analyzed in each of 5 animals per group was limited (7–9 somatic inclusions per series of sections), it was not possible to perform statistical analysis. In mice injected with Ribbons, 98.6% of inclusions in cell soma were found in NeuN-positive neurons, while the rest were in NeuN-negative/Olig2-negative cells (*n* = 5, 11–24 inclusions per series of sections).

Mice injected with strain F-65 mostly formed elongated inclusions, shaped like cellular processes, that did not colocalize with either of our two cell-specific markers. In 4 mice injected with the F-65 strain, we only detected 2 somatic inclusions in total and they were both inside NeuN-positive neurons.

The injection of strain F-91 led to pser129-immunoreactive inclusions (20 inclusions analyzed in each of 5 mice) that all were inside cell bodies of NeuN-positive neurons. Finally, for mice injected with strain F-110, we found 3–12 pser129-immunoreactive inclusions in cell bodies in the AON in each of 5 animals, and also all of these were within NeuN-positive neurons.

In addition, we observed inclusions inside cells shaped like astrocytes in the AON of mice injected with all of the different strains except those injected with strain F-91 (Fig. 6d) (approximately 1 per animal for fibrils and ribbons-injected groups, 1–3 per F-110-injected mouse, and 3–4 per F-65-injected animals). To identify the cell type that these inclusions were present in, we performed GFAP and Iba1 immunofluorescence staining, but we did not identify pser129 inclusions in GFAP-positive cells or in Iba1-positive cells (data not shown). However, it is possible that our analysis of glia overlooked rare instances of pser129 inclusions, because we found relatively few GFAP-positive cells; and in the case of Iba1-positive microglia, autofluorescent lipofuscin granules often hampered detailed analysis. In addition, morphological analysis of Iba-1-positive microglia did not reveal differences in the hydraulic radius of microglia between groups (Additional file 6) indicating no differences in the activation state of microglia between groups.

In conclusion, we detected mature α-syn inclusions after injections of each of the five different α-syn strains, and pser129-immunoreactive inclusions inside cell bodies were mainly localized in neurons. None of the pser129-immunoreactive inclusions we observed were within a cell that was labeled with the oligodendrocyte-marker Olig2.

## Discussion

We assembled monomeric human WT full-length or C-truncated (1–110) α-syn into different fibrillar polymorphs. The assembly was accomplished in a controlled manner and we performed quality control of the different strains before use in vivo (Additional file 1). The quality controls we performed demonstrate that each strain we produced is homogenous, and is consistent in terms of morphology and digestion profiles with strains used in our earlier work [5, 16, 26, 37].

We then precisely micro-injected the different strains unilaterally into the OB of WT mice and kept the mice for 3- or 6-MPI. The injections of different α-syn strains triggered α-synucleinopathy at the injection site as well as in directly- and indirectly-connected brain regions. Our data show α-syn strain strain-dependent differential efficiency in seeding and propagation.

### Distinct strains seeded differently; strain F-91 was the most efficient strain for seeding

The injection of the different strains triggered pser129-positive inclusions at the injection site. No inclusions were observed after injection of α-syn monomers. Based on our earlier work, we know that (exogenous) human α-syn injected into the OB [55] or other brain regions [47] of wild-type rodents becomes undetectable by immunohistochemistry a few days after injection. Thus, the pser129 positive inclusions we detect at 3- and 6-MPI correspond to mouse endogenous α-syn.

Our study is the first to our best knowledge to compare the seeding propensity and spread in vivo of 5 different highly characterized strains (Table 1). We demonstrate here a lower seeding potential of the strains Fibrils and F-65 at the injection site while the strains Ribbons, F-91 and F-110 were very potent. In addition, the strains-F-110 and -F-65-induced inclusions appeared persist over 6 months to a higher extent than those mediated by other strains. To the contrary, the density of Ribbons-induced inclusions declined at 6 MPI. Our results indicate a strong in vivo seeding potential of strain F-91 in particular, and greater seeding potential of Ribbons compared to Fibrils (in agreement with [47]).

**Table 1:**
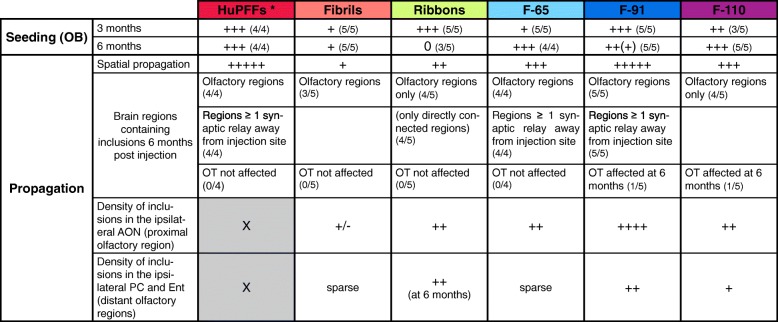
Summary table of propagation of α-syn inclusions observed after injection of different types of fibrils/α-syn strains in the OB of wild type mice

Summary table of the results and comparisons between strains from our study and including previously published data from huPFFs injections [54, 56]. The proportion of animals following the pattern described in the table is indicated in parenthesis

*The huPFFs were injected at higher concentration (5 μg/μL) than the strains (monomers, fibrils, ribbons, F-65, F-91 and F-110) used in this work (2 μg/μL); the volume injected remained the same (0.8 μL, 4 μg of huPFFs versus 1.6 μg of each strain per OB). The density of inclusions in the AON, PC and Ent (grey shaded boxes) after HuPFFs injection is not reported in the table because the analysis was performed separately from the other groups

In our previous studies, we have injected both preformed fibrils made of human (huPFFs) or mouse α-syn (mPFFs) in the OB [54, 56]. Mouse PFFs, possibly because of the lack of a species barrier, induced a more widespread propagation that human PFFs in WT mice [54, 56], which was confirmed by another study [35]. Here, when comparing the huPFFs to our 5 strains also made of human α-syn, we observe that the seeding potential and the propagation pattern induced by huPFFs injection in the OB were very comparable to those of low amounts of the strain F-91 (Fig. 4, Table 1). However, as the huPFFs were not structurally characterized, our results with strain F-91 cannot be directly compared to huPFFs used in earlier studies.

We previously assessed the seeding potential the strains Fibrils and Ribbons *in cellulo* and in vivo [26, 47]. Human induced pluripotent stem cells (hiPSC) differentiated into neurons took up the two strains to similar extents, but Ribbons were more efficient at inducing pathological inclusions over weeks [26]. When injected into the substantia nigra of rats, Ribbons also induced more abundant inclusions than fibrils at the injection site [47]. Our present study strengthens those observations by demonstrating higher seeding potential of Ribbons was compared to Fibrils after injection into the OB of WT mice. Interestingly, we observed less Ribbons-induced pathology at 6 MPI in the OB, compared to 3 months. It is possible that Ribbons triggered cell death or induced a higher neurogenic turnover of OB cells. However, when neurons derived from hiPSC were exposed to Ribbons, no neuronal death was observed [26]. In addition, the injection of Ribbons did not induce loss of dopaminergic neurons in the substantia nigra of WT rats [47], and we saw no reduction in density of cresyl violet-stained cells in the AON 6 MPI (not shown) suggesting no severe cell loss at 6 MPI, making cell loss an unlikely contributor to the decrease inclusions in the OB.

Our study is the first investigating the effect of F-110 strain in vivo. We demonstrate that this strain propagated well in the brain and induced more pathology in OB and AON as compared to strains made of full-length α-syn. We previously demonstrated that the Fibril-strain is processed in neuronal cells yielding a fibrillar α-syn truncated C-terminally at residue 115 [49].

### Each strain triggered propagation of pathology with different kinetics and efficiency

We investigated the spreading of pathology through the neuronal connectome. Our work demonstrated that the spatial spreading pattern of strain-induced inclusions is dependent on the conformation of the injected strain (Table 1). Importantly, pathology that developed in distant regions could result from either the trans-neuronal propagation of the injected assemblies to distant regions, followed by the “on site” seeding of endogenous α-syn; or from the trans-neuronal propagation of endogenous seeds that were templated at the injection site.

Since we cannot determine with certainty a direction of propagation via the neuronal network (anterograde or retrograde), we will focus the discussion on the shorter route of likely propagation. The strains F-65, F-91 and F-110 triggered propagation to brain regions that require crossing at least one synaptic relay. The strain F-91 propagated the furthest, through direct olfactory connections and reached bilateral circuits triggering significant pser129 inclusion load, similar to the propagation pattern of huPFFs in our previous work [54, 56]. F-91-induced inclusions were also detected in distant non-olfactory regions that require crossing at least one synaptic relay. The strain F-65 propagated slightly less and reached a few distant non-olfactory regions. Despite their ability to cross synaptic relays, the F-110-induced inclusions were only seen within olfactory structures. Fibrils-induced inclusions propagated within the olfactory system solely and reached only a limited number of directly connected structures where they triggered relatively limited pathology. The contralateral OT, affected at 6 MPI, is the only structure that requires the crossing of one synaptic relay. Distinctly, Ribbons were the only strain that did not cross one synaptic relay within 6 months. Surprisingly, Ribbons-induced inclusions efficiently reached the ipsilateral Ent, a distant OB-connected region.

Among the strains that propagated to distant olfactory regions, the strains F-91 and Ribbons led to moderate or dense inclusion load, while the strains F-65 and F-110 triggered sparse and thin inclusions that are below detection level when assessed with our densitometry method. When looking at proximal olfactory regions, the strains F-91 and Ribbons were the most efficient at spreading and led to the highest inclusion densities measured at 3 MPI. Ribbons-induced pathology was significantly lower at 6 than 3 months in both the ipsilateral and contralateral AON, suggesting efficient clearing of these inclusions. Strains F-65 and F-110 triggered significant pathology in ipsilateral AON that was greater at 6 than 3 MPI.

Interestingly, the injection of Fibrils, Ribbons and F-65 did not trigger inclusions in the OT at the latest time point although other olfactory regions were already affected, suggesting that the OT is less permissive to seeding with these strains. For the strains F-91 and F-110, inclusions in the OT were detected in one animal out of 5 and appeared 6 months post injection, e.g. 3 months later than in other olfactory regions. In our earlier work with human PFFs, the appearance of inclusions in the OT was delayed to 9 MPI, compared to other olfactory structures [54, 56]. The OT receives direct projections from the OB, but unlike other olfactory structures, it does not project back to the OB. The delay in the development of inclusions in the OT could indicate a lower susceptibility of this structure to develop inclusions or would support a retrograde direction of propagation. We have recently demonstrated using connectome modeling that PFFs-induced pathology preferentially propagates retrogradely during early time points (1- to 9 months post injection) and then shifts to an anterograde progression following longer delays after OB injection [42].

### Characterization of inclusions and of cells carrying strain-induced pathology

The α-syn strains triggered the formation of different types of cellular inclusions. Strain F-65 occasionally triggered somatic inclusions, and also the formation of inclusions in neurites and astrocyte-like processes. Inclusions induced by Fibrils, Ribbons and F-110 strains were predominantly neurite-like inclusions, while strain F-91 most frequently induced inclusions in neuronal soma. All the assemblies triggered Thioflavin-S-positive inclusions indicating mature amyloid structures.

We quantified the proportion of somatic inclusions localized within neurons versus oligodendrocytes in the AON. The AON is the brain region that developed the highest density of inclusions following injection of the different assemblies in the OB. Most inclusions were inside neurons, none were detected in oligodendrocytes. An earlier in vivo study showed inclusions in oligodendrocytes in the substantia nigra after intranigral injection of Ribbons in rats that overexpressed mutant α-syn, but not in similarly injected WT rats [47]. In line with those findings, inoculation of glial cytoplasmic inclusion (GCI)-extracted α-syn triggered oligodendroglial inclusions in KOM2 mice that overexpressed α-syn specifically in oligodendrocytes, but did not trigger inclusions in WT mice [48] or in mice overexpressing α-syn in neurons [4, 13, 64]. The consensus is that the low level of endogenous α-syn expressed in oligodendrocytes and their progenitors [14] is not sufficient to allow the formation of intracellular α-syn inclusions.

### Importance of the characterization of assemblies and of quality controls for comparable and reproducible studies

We have demonstrated here that different pure and homogenous assemblies, made of the same WT α-syn exhibit different seeding and propagation propensities when injected into the brain of WT mice. As intracerebral injections of recombinant assemblies (fibrils, PFFs) are now widely used in the field, it appears crucial that each laboratory characterizes the assemblies they produce and control-check their different batches for homogeneity and consistency with previous batches to the extent presented here. Indeed, different batches of fibrillar α-syn produced by a given laboratory with a defined protocol may vary with additional variability between assemblies generated in different laboratories. We believe that such variations could explain the variability of the histological and behavioral results obtained from different laboratories [31, 33, 36, 38, 39, 44, 45, 56, 58], even upon the use of a given experimental model. We further believe that the homogeneity of α-syn fibrillar polymorphs preparations is critical for reproducibility as it is impossible to attribute an observed effect to a given polymorph within a mixture.

## Conclusion

In summary, we demonstrate that five conformational strains of human α-syn exhibit strain-dependent efficiencies at inducing seeding and propagation of α-syn inclusions in WT mice. Our data support the hypothesis that the polymorphism of fibrillar α-syn could also underlie different propagation patterns of α-syn pathology in humans. The misfolded α-syn present in patients with different synucleinopathies needs to be characterized in more detail in the future, and their properties compared with those of strains generated in vitro from recombinant α-syn. Ultimately this could lead to a precision medicine approach with therapeutic strategies that are tailored to each type of synucleinopathy or even to each individual patient.

## Supplementary information

**Additional file 1.** Quality control of the assemblies.

**Additional file 2.** List of antibodies, references and working concentrations.

**Additional file 3.** Examples of pser129-positive inclusions at high and low magnifications.

**Additional file 4.** List of the abbreviations of brain regions used in the figures.

**Additional file 5.** Negative binomial mixed-effects model analysis of pser129 quantifications.

**Additional file 6. **Microglial morphology analysis reveals no differences in microglial activation between groups.

## Data Availability

The datasets used and/or analyzed during the current study are available from the corresponding author on reasonable request.
