# Supplementary material for: α-Synuclein conformational strains spread, seed and target neuronal cells differentially after injection into the olfactory bulb
Source: Acta Neuropathol Commun. 2019 Dec 30;7:221. doi: 10.1186/s40478-019-0859-3 (PMC6937797; doi:10.1186/s40478-019-0859-3)
Supplement: Supplementary file 1 — Additional file 1. Quality control of the assemblies. [file 40478_2019_859_MOESM1_ESM.pdf]

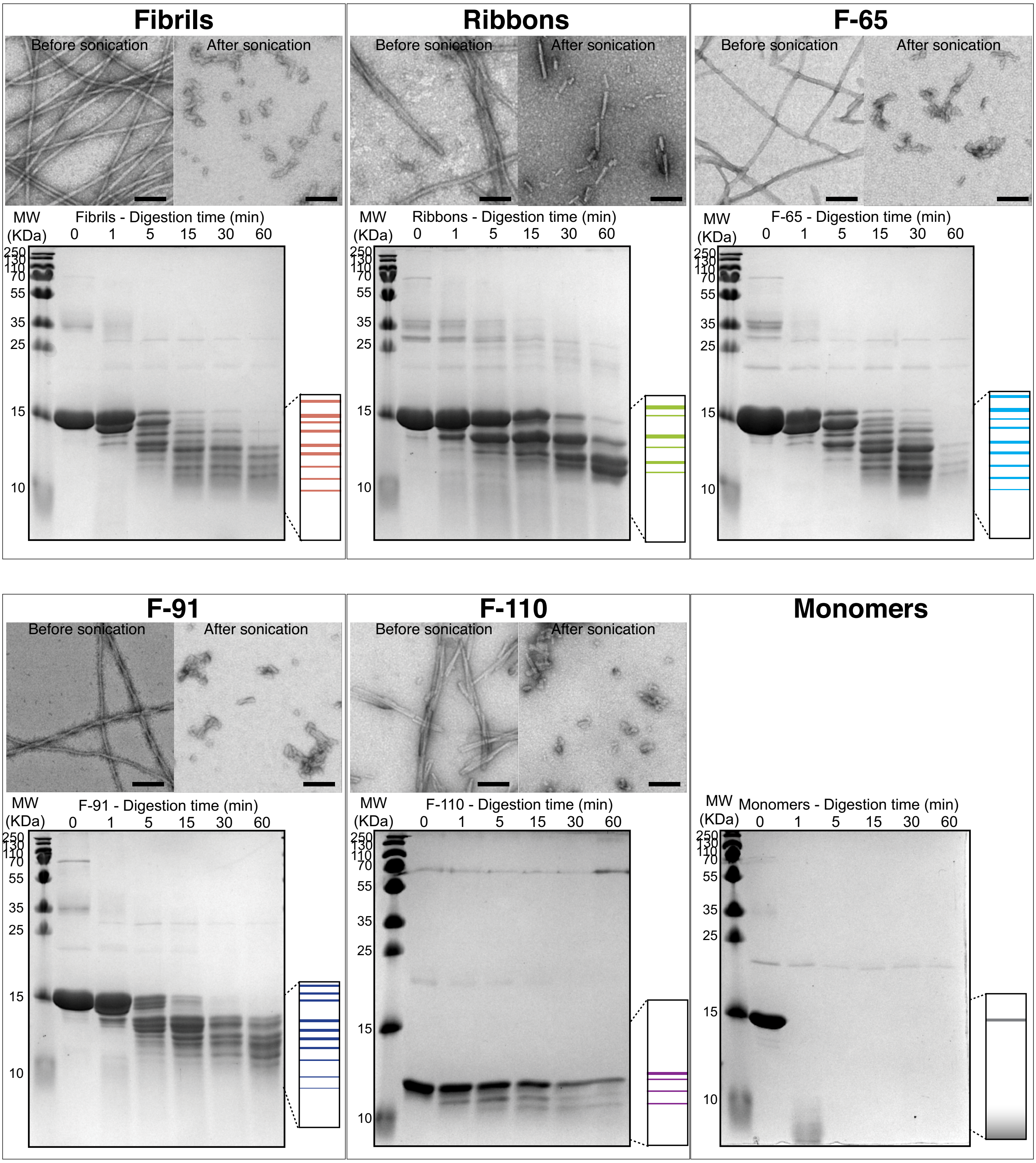

Additional file 1: **Quality control of the assemblies**

The quality of the assemblies was verified by electron microscopy, and by checking limited proteolysis profiles. Transmission electron microscopy (TEM) images of the assemblies were obtained after negative-staining by uranyl acetate. Scale bar: 200 nm

Proteinase-K digested samples collected from 0 to 60 min after addition of protease are visualized after migration on SDS-PAGE (15%) and Coomassie staining. Next to the gels, we represented the proteolysis profile (10 to 15 KDa) as a “barcode” that is specific and unique to each strain.
