# Supplementary material for: α-Synuclein conformational strains spread, seed and target neuronal cells differentially after injection into the olfactory bulb
Source: Acta Neuropathol Commun. 2019 Dec 30;7:221. doi: 10.1186/s40478-019-0859-3 (PMC6937797; doi:10.1186/s40478-019-0859-3)
Supplement: Supplementary file 2 — Additional file 2. List of antibodies, references and working concentrations. [file 40478_2019_859_MOESM2_ESM.pdf]

Antibodies	Reference	Source	Host	Immunohistochemistry	Immunofluorescence	Remark
**Primary antibodies:**						
Phosphorylated alpha-synuclein Ser129 (EP1536Y clone)	Ab51253	Abcam	Rabbit monoclonal	1:10 000	1:10 000 or 1:5 000	
NeuN	MAB377	Millipore	Mouse monoclonal		1:1000	
Olig2-AF488 (batch 2897028)	AB9610-AF488	Millipore	Rabbit polyclonal		1:500	
Iba1	Ab5076	Abcam	Goat polyconal		1:500	
GFAP	MAB360	Millipore	Mouse monoclonal		1:500	
**Secondary antibodies:**						
Goat anti-mouse A633	A21050	Life Technologies			1:500	Olig2/Pser129/NeuN staining
Goat anti-rabbit A568	A-11011	Molecular Probes			1:500	Olig2/Pser129/NeuN staining
Goat anti-mouse A594	115-585-166	Jackson Immunoresearch			1:400	ThS/ NeuN Staining
Donkey anti-goat A488					1:500	Iba1/Pser129/GFAP
Donkey anti-rabbit A594					1:500	Iba1/Pser129/GFAP
Donkey anti-mouse A680					1:500	Iba1/Pser129/GFAP
Goat anti-rabbit biotinlylated	BA-1000	Vector Labs		1:500		Pser129 staining

Additional file 2: List of antibodies, references and working concentrations
