# Supplementary material for: α-Synuclein conformational strains spread, seed and target neuronal cells differentially after injection into the olfactory bulb
Source: Acta Neuropathol Commun. 2019 Dec 30;7:221. doi: 10.1186/s40478-019-0859-3 (PMC6937797; doi:10.1186/s40478-019-0859-3)
Supplement: Supplementary file 3 — Additional file 3. Examples of pser129-positive inclusions at high and low magnifications. [file 40478_2019_859_MOESM3_ESM.pdf]

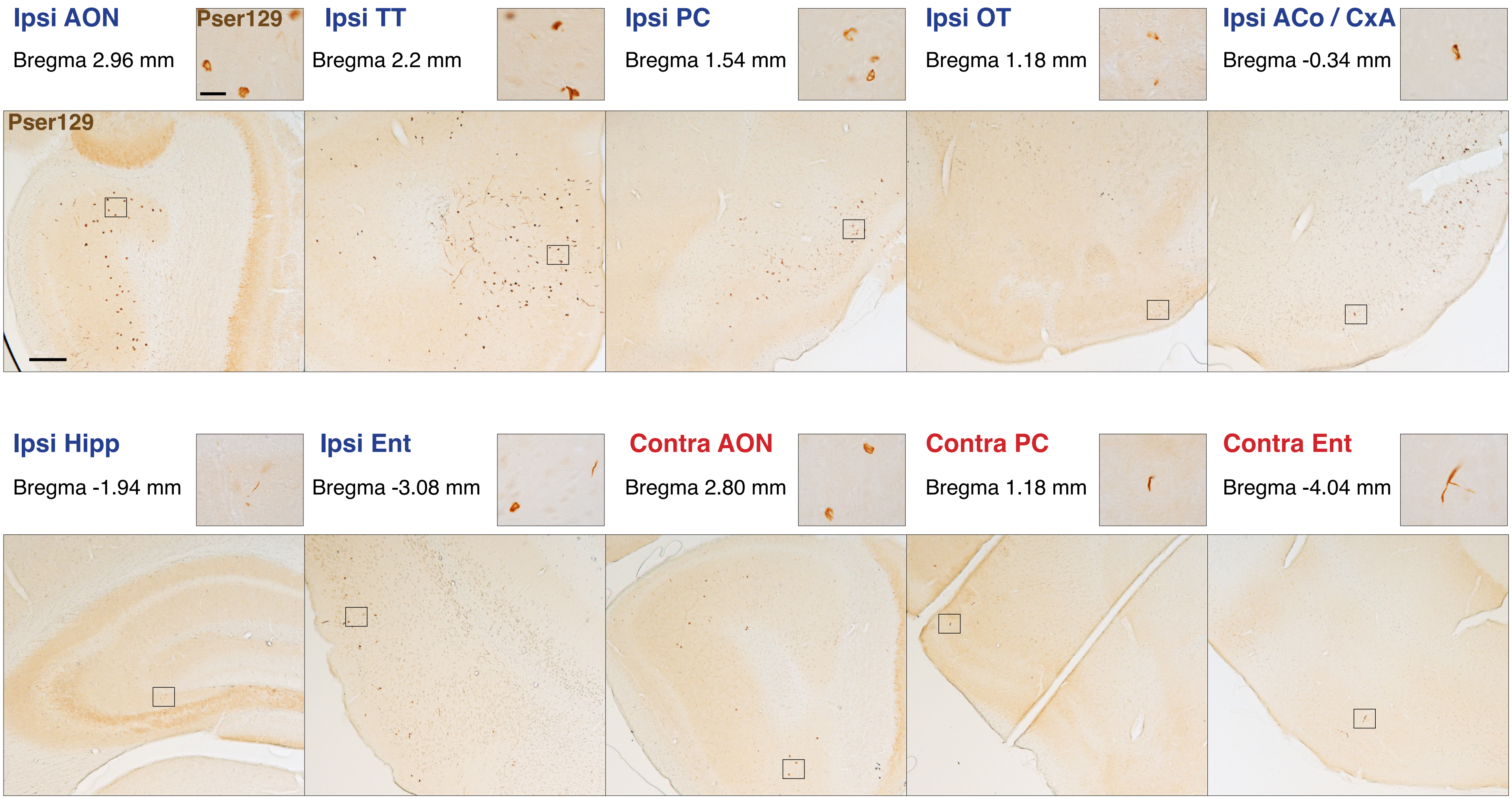

Additional file 3: **Examples of pser129-positive inclusions at high and low magnifications**
Examples of photomicrographs from an animal injected with F-91 in the ipsi OB showing alpha-synulclein inclusions in some ipsilateral (legend in blue) and contralateral (legend in red) brain regions at 6 months post injection (5x and 63x magnification). Pser129-positive inclusions were detected by an antibody directed against alpha-synuclein phosphorylated on serine 129 (pser129). The area observed at 63x is identified on the low magnification image by a black rectangle. A list of brain structure abbreviations is provided as additional file 4.
Scale bar: Low magnification images: 200 µm; High magnification images: 25 µm
