# Supplementary material for: α-Synuclein conformational strains spread, seed and target neuronal cells differentially after injection into the olfactory bulb
Source: Acta Neuropathol Commun. 2019 Dec 30;7:221. doi: 10.1186/s40478-019-0859-3 (PMC6937797; doi:10.1186/s40478-019-0859-3)
Supplement: Supplementary file 4 — Additional file 4. List of the abbreviations of brain regions used in the figures. [file 40478_2019_859_MOESM4_ESM.pdf]

Abbreviation	Structure name
AcbNu	Accumbens Nucleus Core and Shell
aca	Anterior part of the anterior commissure
aci	Anterior part of the anterior commissure, intrabulbar part
ACo	Anterior cortical nucleus of the amygdala
AHiPM, AHiAL	Amygdalohippocampal area posteromedial, part anterolateral part
Am	Amygdala (group all the amygdaloid nuclei)
APir	Amygdalopiriform transition area
AON	Anterior olfactory nucleus
AOB	Accessory olfactory bulb
AuV, AuD	Secondary auditory cortex
BLA, BLV, BLP	Basal amygdaloid nucleus
BMA, BMP, BML	Accessory basal amygdaloid nucleus (Amygdala)
CeL, CeM, CeC	Central amygdaloid nucleus (Amygdala), Lateral, Medial, Central
Cg	Cingulate cortex
CPu	Caudate putamen
CxA	Cortex amygdala transition area
DEn, IEn	Dorsal endopiriform nucleus, Immediate endopiriform Nucleus
DMX	Dorsal motor nucleus of the vagus nerve
DP	Dorsal peduncular cortex
Ect	Ectorhinal cortex
Ent	Entorhinal cortex
E/OV	Ependymal and subependymal layer/olfactory ventricle
FC	Frontal cortex and orbital cortex
Hipp	Hippocampus
Hipp: Rad	Radial layer of the hippocampus
Hipp: Mol	Molecular layer of the hippocampus
Hipp: Pyr	Pyramidal layer of the hippocampus
Hipp: DG	Dentate gyrus of the hippocampus
Hipp: CA	Cornu Ammonis of the hippocampus
Hipp: Or	Oriens layer of the hippocampus
Hth (PLH, LH)	Lateral hypothalamic area
Ins	Insular cortex
LC	Locus Coeruleus
LSI	Lateral septal nucleus
M2	Secondary motor cortex
MeAD, MePV	Medial Nucleus of the amygdala
MOB	Main olfactory bulb
mVeMC, mVePC	Medial vestibular nucleus, magnocellular part, parvicellular part
nLOT	Nucleus of the lateral olfactory tract
OB	Olfactory bulb
OT	Olfactory tubercle
PC	Piriform cortex
PLCo	Posterolateral cortical amygdaloid area
PMCo	Posteromedial cortical amygdaloid area
PRh	Perirhinal cortex
RN	Dorsal and medial raphe nuclei
S1	Primary somatosensory cortex
S2	Secondary somatosensory cortex
SN, SNR, SNpc	Substantia nigra, reticulata, pars compacta
STMAM, STMPM	Antero medial part of the bed nucleus of the stria terminalis
STr	Subiculum transition area
TeA	Temporal cortex association area
Th	Thalamus
TT	Tenia tecta
VS	Ventral subiculum
VP	Ventral pallidum
VTA	Ventral tegmental area

Additional file 4: List of the abbreviations of brain regions used in the figures
