# Supplementary material for: α-Synuclein conformational strains spread, seed and target neuronal cells differentially after injection into the olfactory bulb
Source: Acta Neuropathol Commun. 2019 Dec 30;7:221. doi: 10.1186/s40478-019-0859-3 (PMC6937797; doi:10.1186/s40478-019-0859-3)
Supplement: Supplementary file 5 — Additional file 5. Negative binomial mixed-effects model analysis of pser129 quantifications. [file 40478_2019_859_MOESM5_ESM.pdf]

Additional file 5

a.

Brain region	Side	Linear mixed effect model	3 mo Monomers/Fibrils	3 mo Monomers/Ribbons	3 mo Monomers/F-65	3 mo Monomers/F-91	3 mo Monomers/F-110	6 mo Monomers/Fibrils	6 mo Monomers/Ribbons	6 mo Monomers/F-65	6 mo Monomers/F-91	6 mo Monomers/F-110
OB	Ipsi-lateral	M. Estim.	-2.2024159	-4.3856491	-2.085004	-4.0819228	-3.2316901	-3.2979225	-0.4531421	-5.3266885	-4.0411989	-5.3175606
		SE	0.8050185	0.7986151	0.793322	0.893377	0.7936244	0.7590524	0.825564	0.7715956	0.7563192	0.7553405
		p-value	0.0155545 *	0.0000006 ***	0.0183942 *	0.0000367 ***	0.000233 ***	0.0000232 ***	0.6247311	<10^-8***	0.0000002 ***	<10^-8***
	Contra-lateral	M. Estim.	1.03037	-0.5297737	-0.0070598	0.1753733	-0.1943579	2.2245383	2.5565845	-1.6977794	0.9053057	-2.0349129
		SE	0.9992718	0.8915931	0.8904079	0.9025464	0.8870308	1.3980694	1.6382832	0.7768376	0.9480855	0.7492252
		p-value	0.9063575	0.9063575	0.9936739	0.9063575	0.9063575	0.1779532	0.1779532	0.0540978	0.4039909	0.0141581 *
AON	Ipsi-lateral	M. Estim.	-0.9866532	-4.4896029	-2.7947744	-6.0413418	-3.1317466	-2.396617	-3.1240166	-5.0501018	-5.8524287	-5.4593612
		SE	1.2564714	1.2139637	1.1967463	1.174415	1.189928	0.7472861	0.7391925	0.7443683	0.7360689	0.7302891
		p-value	0.4631816	0.0010852 **	0.0366135 *	0.000004 ***	0.0181959 *	0.0018284 **	0.0000396 ***	0.0000002 ***	0.0000002 ***	0.0000002 ***
	Contra-lateral	M. Estim.	-0.9866532	-4.4896029	-2.7947744	-6.0413418	-3.1317466	-2.396617	-3.1240166	-5.0501018	-5.8524287	-5.4593612
		SE	1.2564714	1.2139637	1.1967463	1.174415	1.189928	0.7472861	0.7391925	0.7443683	0.7360689	0.7302891
		p-value	0.4631816	0.0010852 **	0.0366135 *	0.000004 ***	0.0181959 *	0.0018284 **	0.0000396 ***	0.0000002 ***	0.0000002 ***	0.0000002 ***
PC	Ipsi-lateral	M. Estim.	1.2411292	-0.5977978	-0.8412348	-2.7794372	-0.2825007	-1.2085752	-3.8674977	-2.7049067	-6.5374704	-3.3238775
		SE	1.0148844	0.687415	0.6706483	0.6156702	0.7152628	1.3274571	1.2441636	1.2758071	1.2369872	1.2492732
		p-value	0.3320361	0.4806272	0.3320361	0.0000238 ***	0.6928719	0.3625885	0.0040293 **	0.0509897	0.0000004 ***	0.0129984 *
	Contra-lateral	M. Estim.	0.7466176	-1.0942839	-0.7207084	-0.8073693	-0.0529057	-1.2085752	-3.8674977	-2.7049067	-6.5374704	-3.3238775
		SE	1.0389776	0.7593502	0.7871265	0.7798261	0.8662943	1.3274571	1.2441636	1.2758071	1.2369872	1.2492732
		p-value	0.644158	0.4486868	0.5997774	0.5997774	0.9513025	0.3625885	0.0040293 **	0.0509897	0.0000004 ***	0.0129984 *
Ent	Ipsi-lateral	M. Estim.	2.3777117	0.059488	-0.2948478	-2.9023137	0.0656126	0.6199824	-1.5764212	-0.0973174	-4.7521721	0.2148429
		SE	2.2358394	1.1177328	1.0465886	0.9030662	1.1035645	1.1013609	0.8819471	1.0190183	0.8584345	1.0129215
		p-value	0.4931576	0.9954183	0.9726951	0.0049114 **	0.9954183	0.7820273	0.1231126	0.9239168	0.0000001 ***	0.8914578
	Contra-lateral	M. Estim.	1.6500186	-0.3383838	-0.3304787	-0.4803679	-0.4522638	0.6199824	-1.5764212	-0.0973174	-4.7521721	0.2148429
		SE	1.3685588	0.9035141	0.8866803	0.8670781	0.8752876	1.1013609	0.8819471	1.0190183	0.8584345	1.0129215
		p-value	0.6838441	0.9921703	0.9921703	0.9921703	0.9921703	0.7820273	0.1231126	0.9239168	0.0000001 ***	0.8914578

Additional file 5a: Negative binomial mixed-effects model analysis of pser129 quantifications

Comparison of the control group (monomers) versus each strain within same side and same delay post-injection.
Analyses for each brain regions were performed separately but gathered on the same table for easier reading.
M. Estim. = Model estimate *: $p<0.05$, **: $p<0.01$, ***: $p<0.001$ Comparison to monomers

Additional file 5

b.

Brain region	Side	Linear mixed effect model	3 mo Fibrils/Ribbons	Fibrils/F-65	Fibrils/F-91	Fibrils/F-110	Ribbons/F-65	Ribbons/F-91	Ribbons/F-110	F-65/F-91	F-65/F-110	F-91/F-110
OB	Ipsi-lateral	M. Estim.	-2.1832332	0.1174119	-1.8795069	-1.0292742	2.3006451	0.3037263	1.153959	-1.9969188	-1.1466862	0.8502327
		SE	0.7385211	0.7205043	0.7989042	0.7298316	0.7186134	0.8683246	0.7059378	0.810357	0.7120473	0.8499336
		p-value	0.009343 ##	0.8705515	0.0310706 #	0.1980676	0.0051267 ##	0.7783935	0.1463304	0.0257441 #	0.1463304	0.365931
	Contra-lateral	M. Estim.	-1.5601436	-1.0374297	-0.8549967	-1.2247279	0.5227139	0.7051469	0.3354158	0.182433	-0.1872981	-0.3697311
		SE	0.9484891	0.9552479	0.9651527	0.9470998	0.8456158	0.8526187	0.8213516	0.8523138	0.8386007	0.8481842
		p-value	0.9063575	0.9063575	0.9063575	0.9063575	0.9063575	0.9063575	0.9063575	0.9063575	0.9063575	0.9063575
AON	Ipsi-lateral	M. Estim.	-3.5029497	-1.8081212	-5.0546887	-2.1450934	1.6948285	-1.5517389	1.3578563	-3.2465674	-0.3369722	2.9095952
		SE	1.0831337	1.0554826	1.0374847	1.0487138	1.0057821	0.9672726	0.9968375	0.9564656	0.9760711	0.9478167
		p-value	0.003661 ##	0.1254166	0.0000083 ###	0.0680172	0.1254166	0.135826	0.1997839	0.0025798 ##	0.729919	0.0053553 ##
	Contra-lateral	M. Estim.	-3.5029497	-1.8081212	-5.0546887	-2.1450934	1.6948285	-1.5517389	1.3578563	-3.2465674	-0.3369722	2.9095952
		SE	1.0831337	1.0554826	1.0374847	1.0487138	1.0057821	0.9672726	0.9968375	0.9564656	0.9760711	0.9478167
		p-value	0.003661 ##	0.1254166	0.0000083 ###	0.0680172	0.1254166	0.135826	0.1997839	0.0025798 ##	0.729919	0.0053553 ##
PC	Ipsi-lateral	M. Estim.	-1.838927	-2.0823641	-4.0205664	-1.5236299	-0.2434371	-2.1816394	0.3152971	-1.9382023	0.5587341	2.4969365
		SE	0.9389844	0.9267801	0.8878075	0.9595589	0.5490632	0.4803655	0.6027455	0.4560491	0.5835511	0.5194349
		p-value	0.1075298	0.0616192	0.0000238 ###	0.210603	0.6928719	0.0000238 ###	0.6928719	0.0000641 ###	0.4613575	0.000023 ###
	Contra-lateral	M. Estim.	-1.8409015	-1.467326	-1.5539869	-0.7995232	0.3735755	0.2869146	1.0413782	-0.0866609	0.6678028	0.7544637
		SE	0.8977847	0.9213971	0.9151685	0.989884	0.588312	0.5785081	0.6906582	0.6145136	0.7210853	0.7131092
		p-value	0.4486868	0.4486868	0.4486868	0.6289003	0.6567892	0.7152986	0.4486868	0.9512693	0.5997774	0.5997774
Ent	Ipsi-lateral	M. Estim.	-2.3182237	-2.6725594	-5.2800254	-2.3120991	-0.3543358	-2.9618017	0.0061246	-2.6074659	0.3604604	2.9679263
		SE	2.2178061	2.1826184	2.1174412	2.2103014	1.0072664	0.8573614	1.0665577	0.7609389	0.9909495	0.8371119
		p-value	0.4931576	0.4931576	0.0379378 #	0.4931576	0.9726951	0.0030555 ##	0.9954183	0.0030555 ##	0.9726951	0.0030555 ##
	Contra-lateral	M. Estim.	-1.9884023	-1.9804973	-2.1303865	-2.1022824	0.007905	-0.1419842	-0.1138801	-0.1498892	-0.1217851	0.0281041
		SE	1.3175912	1.3055816	1.2916974	1.2975723	0.805541	0.7843568	0.7931928	0.7637672	0.7731684	0.7495432
		p-value	0.492256	0.492256	0.492256	0.492256	0.9921703	0.9921703	0.9921703	0.9921703	0.9921703	0.9921703

Brain region	Side	Linear mixed effect model	6 mo Fibrils/Ribbons	Fibrils/F-65	Fibrils/F-91	Fibrils/F-110	Ribbons/F-65	Ribbons/F-91	Ribbons/F-110	F-65/F-91	F-65/F-110	F-91/F-110
OB	Ipsi-lateral	M. Estim.	2.8447804	-2.0287661	-0.7432764	-2.0196382	-4.8735464	-3.5880568	-4.8644185	1.2854896	0.0091279	-1.2763617
		SE	0.576746	0.493468	0.4678691	0.4634823	0.5941347	0.5736232	0.5711937	0.4893055	0.4870006	0.4605115
		p-value	0.0000017 ###	0.000059 ###	0.1293938	0.0000232 ###	<10^-8 ###	<10^-8 ###	<10^-8 ###	0.0107622 #	0.985046	0.0076062 ##
	Contra-lateral	M. Estim.	0.3320463	-3.9223177	-1.3192326	-4.2594512	-4.2543639	-1.6512788	-4.5914975	2.6030851	-0.3371336	-2.9402186
		SE	1.9423776	1.3011758	1.4119315	1.2842375	1.5556597	1.6501344	1.5409011	0.7998789	0.543433	0.7735782
		p-value	0.8642644	0.0086546 ##	0.4039909	0.0056826 ##	0.0141581 #	0.4039909	0.0086546 ##	0.0056826 ##	0.5732243	0.0021634 ##
AON	Ipsi-lateral	M. Estim.	-0.7273996	-2.6534849	-3.4558117	-3.0627442	-1.9260852	-2.7284121	-2.3353446	-0.8023268	-0.4092593	0.3930675
		SE	0.466607	0.478967	0.4649651	0.4582404	0.4635965	0.4461244	0.4411165	0.4458627	0.4426433	0.4166226
		p-value	0.1373273	0.0000001 ###	<10^-8 ###	<10^-8 ###	0.0000489 ###	<10^-8 ###	0.0000002 ###	0.0899259	0.3551842	0.3551842
	Contra-lateral	M. Estim.	-0.7273996	-2.6534849	-3.4558117	-3.0627442	-1.9260852	-2.7284121	-2.3353446	-0.8023268	-0.4092593	0.3930675
		SE	0.466607	0.478967	0.4649651	0.4582404	0.4635965	0.4461244	0.4411165	0.4458627	0.4426433	0.4166226
		p-value	0.1373273	0.0000001 ###	<10^-8 ###	<10^-8 ###	0.0000489 ###	<10^-8 ###	0.0000002 ###	0.0899259	0.3551842	0.3551842
PC	Ipsi-lateral	M. Estim.	-2.6589225	-1.4963315	-5.3288952	-2.1153023	1.1625909	-2.6699728	0.5436202	-3.8325637	-0.6189708	3.2135929
		SE	0.6992946	0.7524565	0.6846609	0.7060305	0.5924052	0.5015817	0.5347303	0.5727658	0.6025061	0.5141692
		p-value	0.0003584 ###	0.0621314	<10^-8 ###	0.0051282 ##	0.0621314	0.0000004 ###	0.331427	<10^-8 ###	0.331427	<10^-8 ###
	Contra-lateral	M. Estim.	-2.6589225	-1.4963315	-5.3288952	-2.1153023	1.1625909	-2.6699728	0.5436202	-3.8325637	-0.6189708	3.2135929
		SE	0.6992946	0.7524565	0.6846609	0.7060305	0.5924052	0.5015817	0.5347303	0.5727658	0.6025061	0.5141692
		p-value	0.0003584 ###	0.0621314	<10^-8 ###	0.0051282 ##	0.0621314	0.0000004 ###	0.331427	<10^-8 ###	0.331427	<10^-8 ###
Ent	Ipsi-lateral	M. Estim.	-2.1964036	-0.7172999	-5.3721545	-0.4051395	1.4791037	-3.1757509	1.7912641	-4.6548547	0.3121604	4.967015
		SE	0.8551436	0.9964072	0.8338673	0.989958	0.7428165	0.5107387	0.7330748	0.7207844	0.8960378	0.7133664
		p-value	0.0255377 #	0.7073925	<10^-8 ###	0.8394876	0.0871083	<10^-8 ###	0.0311696 #	<10^-8 ###	0.8394876	<10^-8 ###
	Contra-lateral	M. Estim.	-2.1964036	-0.7172999	-5.3721545	-0.4051395	1.4791037	-3.1757509	1.7912641	-4.6548547	0.3121604	4.967015
		SE	0.8551436	0.9964072	0.8338673	0.989958	0.7428165	0.5107387	0.7330748	0.7207844	0.8960378	0.7133664
		p-value	0.0255377 #	0.7073925	<10^-8 ###	0.8394876	0.0871083	<10^-8 ###	0.0311696 #	<10^-8 ###	0.8394876	<10^-8 ###

Additional file 5b : Negative binomial mixed-effects model analysis of pser129 quantifications

Comparison between strains, within same side and same delay post-injection.
Analyses for each brain regions were performed separately but gathered on the same table for easier reading.
M. Estim. = Model estimate   #: p<0.05, ##: p<0.01, ###: p<0.001 Strain "x" versus strain "y"

Additional file 5

c.

Brain region	Linear mixed effect model	3 mo						6 mo					
		Fibrils	Ribbons	F-65	F-91	F-110	Monomers	Fibrils	Ribbons	F-65	F-91	F-110	Monomers
OB	M. Estim.	-4.4649874	-4.388424	-3.16558	-4.7005874	-1.221744	-0.4002226	-4.4649874	-4.388424	-3.16558	-4.7005874	-1.221744	-0.4002226
	SE	0.6298229	0.6370201	0.3336784	0.457495	0.3993664	0.3665117	-7.0892745	-6.8889881	0.3336784	0.457495	0.3993664	0.3665117
	p-value	<10^-8 ^^^	<10^-8 ^^^	<10^-8 ^^^	<10^-8 ^^^	0.0022192 ^^	0.3298115	<10^-8 ^^^	<10^-8 ^^^	<10^-8 ^^^	<10^-8 ^^^	0.0022192 ^^	0.3298115
AON	M. Estim.	1.6761565	-2.5189703	-2.1594786	-1.7391823	-1.6485623	-0.5517779	-3.0581055	-2.5189703	-2.1594786	-1.7391823	-2.6730646	-0.5517779
	SE	0.611445	0.4883028	0.3755493	0.1757318	0.4596633	0.7387414	0.4686253	0.4883028	0.3755493	0.1757318	0.3859856	0.7387414
	p-value	0.0122392 ^	0.0000007 ^^^	<10^-8 ^^^	<10^-8 ^^^	0.0005028 ^^^	0.5845694	<10^-8 ^^^	0.0000007 ^^^	<10^-8 ^^^	<10^-8 ^^^	<10^-8 ^^^	0.5845694
PC	M. Estim.	0.4241	0.1054041	-1.2122545	-2.5913344	-1.7215915	-0.3446872	0.4241	-3.7196027	-1.2122545	-2.5913344	-1.7215915	-0.3446872
	SE	0.735694	0.4474629	0.4423505	0.2673365	0.386912	0.7252227	0.735694	0.7502144	0.4423505	0.2673365	0.386912	0.7252227
	p-value	0.7554109	0.8137746	0.0184046 ^	<10^-8 ^^^	0.0000258 ^^^	0.6345841	0.7554109	0.0000043 ^^^	0.0184046 ^	<10^-8 ^^^	0.0000258 ^^^	0.6345841
Ent	M. Estim.	0.7377458	0.8630084	0.0240219	-1.9438931	1.022705	0.2941345	0.7377458	-4.3287253	0.0240219	-1.4071241	-1.4428165	0.2941345
	SE	1.181658	0.9131149	0.5718337	0.789314	0.7502521	0.7291347	1.181658	1.7233015	0.5718337	0.6976	1.3116604	0.7291347
	p-value	0.6388922	0.3445947	0.9664919	0.0206807 ^	0.4774759	0.8239829	0.6388922	0.0316422 ^	0.9664919	0.052423	0.4774759	0.8239829

Additional file 5c : Negative binomial mixed-effects model analysis of pser129 quantifications

Comparison between ipsilateral and contralateral sides, within same experimental group and same delay post-injection.
Analyses for each brain regions were performed separately but gathered on the same table for easier reading.
M. Estim. = Model estimate ^: p<0.05, ^^: p<0.01, ^^^: p< 0.001

Additional file 5

d.

Brain	Side	Linear mixed	Fibrils	Ribbons	F-65	F-91	F-110	Monomers
OB	Ipsi-lateral	M. Estim.	0.3714853	4.7488417	-2.129329	0.6295867	-1.221744	0.9050428
		SE	0.4867145	0.6578444	0.6821667	0.7203321	0.3993664	0.7844643
		p-value	0.4453138	<10^-8 +++	0.0021598 ++	0.3821058	0.0022192 ++	0.3298115
	Contra-lateral	M. Estim.	0.3714853	4.7488417	-2.129329	0.6295867	-1.221744	0.9050428
		SE	0.4867145	0.6578444	0.6821667	0.7203321	0.3993664	0.7844643
		p-value	0.4453138	<10^-8 +++	0.0021598 ++	0.3821058	0.0022192 ++	0.3298115
AON	Ipsi-lateral	M. Estim.	-4.0933411	0.7721403	-2.8849712	-0.1919962	-3.268096	-0.4970839
		SE	1.2232253	0.4864947	1.085671	0.346961	0.8738173	0.7153697
		p-value	0.0024565 ++	0.1124786	0.0094519 ++	0.5800133	0.000368 +++	0.5845694
	Contra-lateral	M. Estim.	0.6409208	0.7721403	-2.8849712	-0.1919962	-2.2435938	-0.4970839
		SE	1.2000682	0.4864947	1.085671	0.346961	0.9178861	0.7153697
		p-value	0.5932928	0.1124786	0.0094519 ++	0.5800133	0.0174158 +	0.5845694
PC	Ipsi-lateral	M. Estim.	-0.3525761	-1.9057367	0.0935708	-2.245044	-1.2519574	1.8395962
		SE	0.730852	0.7224115	0.4096843	0.5058699	0.576653	1.1583737
		p-value	0.7554109	0.0236265 +	0.8193373	0.0000109 +++	0.0359105 +	0.2245341
	Contra-lateral	M. Estim.	-0.3525761	1.9192701	0.0935708	-2.245044	-1.2519574	1.8395962
		SE	0.730852	1.0234814	0.4096843	0.5058699	0.576653	1.1583737
		p-value	0.7554109	0.0911402	0.8193373	0.0000109 +++	0.0359105 +	0.2245341
Ent	Ipsi-lateral	M. Estim.	-0.7768293	-2.4748262	0.5974943	-2.0502804	-0.2423615	0.4628673
		SE	1.190035	0.8311743	0.627415	0.7038957	0.9765097	0.7727112
		p-value	0.6388922	0.0174363 +	0.7547947	0.007165 ++	0.8039862	0.8239829
	Contra-lateral	M. Estim.	-0.7768293	2.7169075	0.5974943	-2.5870494	2.22316	0.4628673
		SE	1.190035	1.7642835	0.627415	0.7837047	1.3233741	0.7727112
		p-value	0.6388922	0.185359	0.7547947	0.0028897 ++	0.4774759	0.8239829

Additional file 5: Negative binomial mixed-effects model analysis of pser129 quantifications

Comparison between 3- and 6-months delays, within same experimental group and same side of the brain.

Analyses for each brain regions were performed separately but gathered on the same table for easier reading.

M. Estim. = Model estimate   +: $p<0.05$, ++: $p<0.01$, +++: $p<0.001$
