# Supplementary material for: α-Synuclein conformational strains spread, seed and target neuronal cells differentially after injection into the olfactory bulb
Source: Acta Neuropathol Commun. 2019 Dec 30;7:221. doi: 10.1186/s40478-019-0859-3 (PMC6937797; doi:10.1186/s40478-019-0859-3)
Supplement: Supplementary file 6 — Additional file 6. Microglial morphology analysis reveals no differences in microglial activation between groups. [file 40478_2019_859_MOESM6_ESM.pdf]

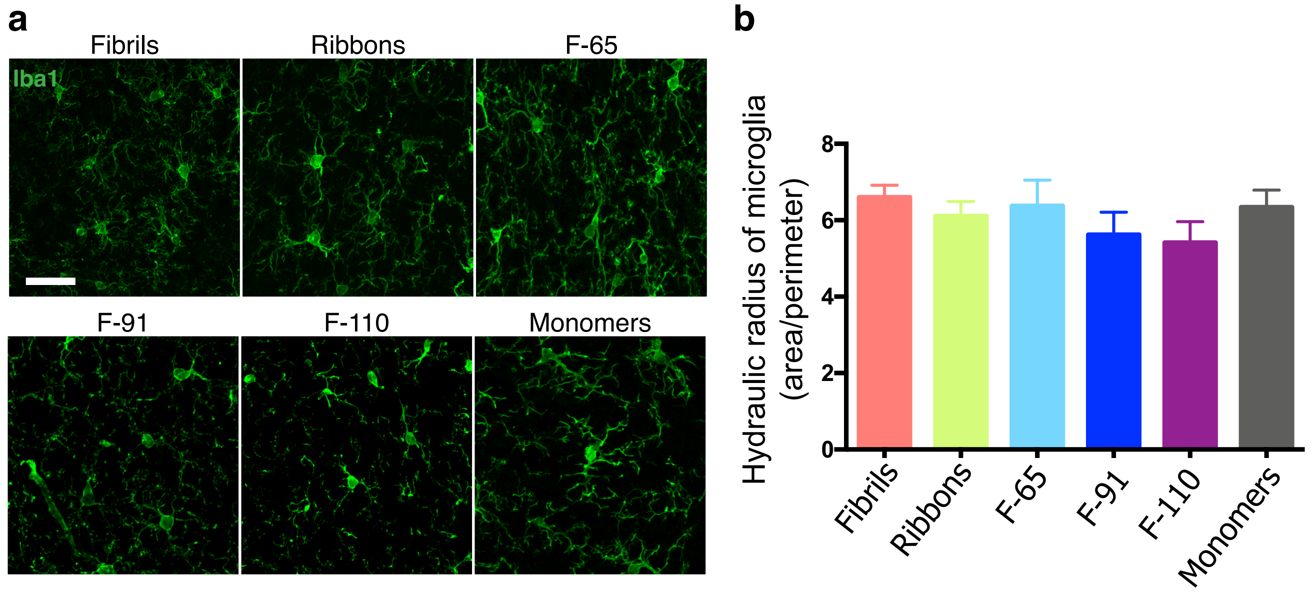

Additional file 6

Microglial morphology analysis reveals no differences in microglial activation between groups.

a. Representative confocal images of Iba1-positive microglia in the OB (maximal projection).

b. Microglia morphology analysis (hydraulic radius: area/perimeter). N= 4 per group; Error bars represent SEM. No significant differences were observed between groups. Scale bar = 10 µm.
